# Structural and functional insights underlying recognition of histidine phosphotransfer protein in fungal phosphorelay systems

**DOI:** 10.1038/s42003-024-06459-0

**Published:** 2024-07-04

**Authors:** Francisco Paredes-Martínez, Lluís Eixerés, Sara Zamora-Caballero, Patricia Casino

**Affiliations:** 1 Departamento de Bioquímica y Biología Molecular, Universitat de València, Burjassot, Spain; 2 Instituto Universitario en Biotecnología y Biomedicina (BIOTECMED), Universitat de València, Burjassot, Spain; 3 Instituto de Biomedicina de Valencia, Consejo Superior de Investigaciones Científicas (IBV-CSIC), Valencia, Spain; 4 CIBER de Enfermedades Raras (CIBERER-ISCIII), Madrid, Spain

**Keywords:** Structural biology, Biochemistry

## Abstract

In human pathogenic fungi, receiver domains from hybrid histidine kinases (hHK) have to recognize one HPt. To understand the recognition mechanism, we have assessed phosphorelay from receiver domains of five hHKs of group III, IV, V, VI, and XI to HPt from *Chaetomium thermophilum* and obtained the structures of Ct_HPt alone and in complex with the receiver domain of hHK group VI. Our data indicate that receiver domains phosphotransfer to Ct_HPt, show a low affinity for complex formation, and prevent a Leu-Thr switch to stabilize phosphoryl groups, also derived from the structures of the receiver domains of hHK group III and *Candida albicans* Sln1. Moreover, we have elucidated the envelope structure of *C. albicans* Ypd1 using small-angle X-ray scattering which reveals an extended flexible conformation of the long loop αD–αE which is not involved in phosphotransfer. Finally, we have analyzed the role of salt bridges in the structure of Ct_HPt alone.

## Abstract (web summary)

Studies on the phosphorelay mechanism from receiver domains of fungal hybrid histidine kinases to HPt from *Chaetomium thermophilum*, including from *Candida albicans*, suggest low affinity for complex formation and absence of a Leu-Thr switch.

## Introduction

**Fig. 1:**
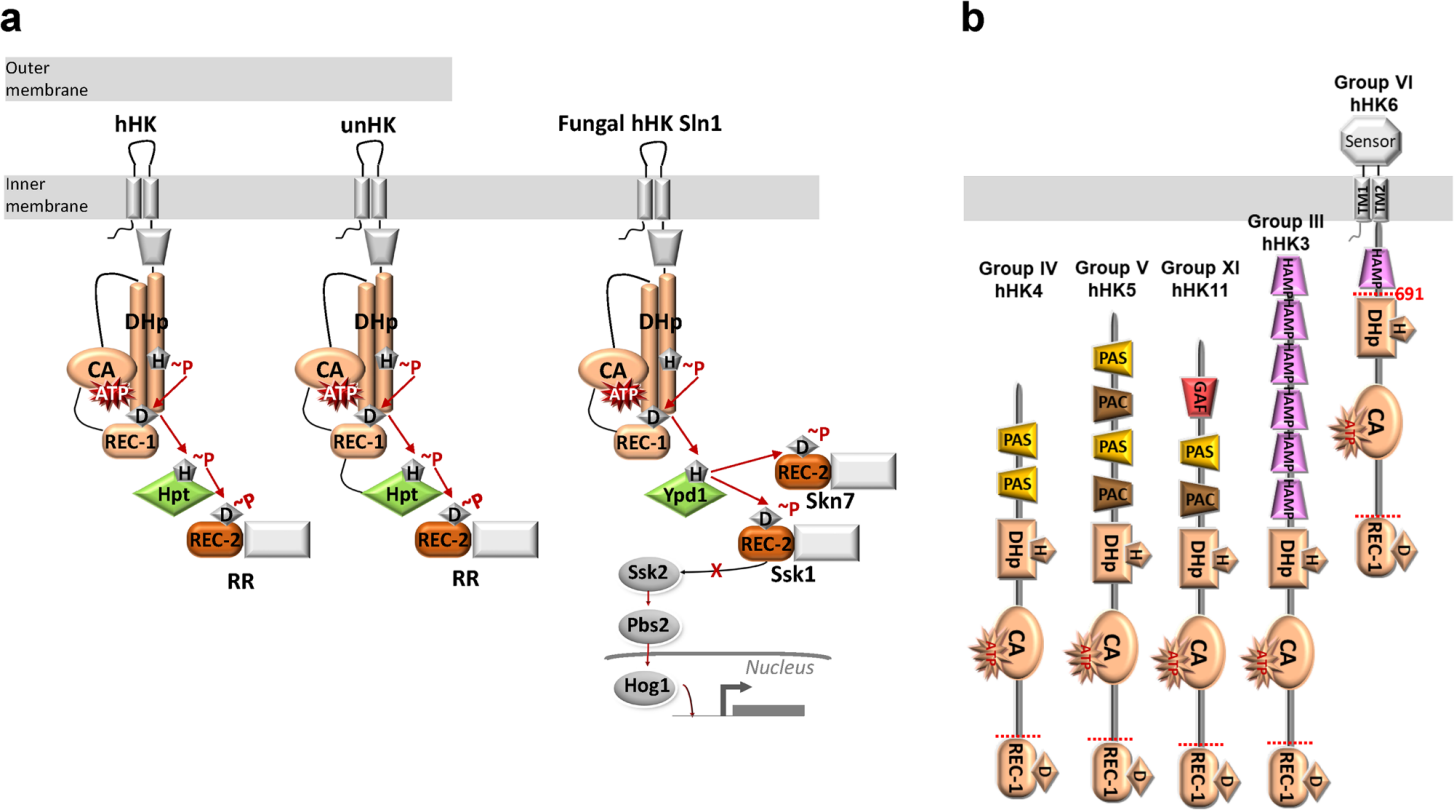
Schematic representation of the role of HPt in microbial signal transduction systems and hHKs from *Chaetomium thermophilum.* **a** Example of domain configuration of hHK and unHK containing transmembrane regions connected to extracellular and intracellular sensor domains (colored in gray). The cytoplasmic catalytic region (in light orange) is comprised of the dimerization His-phosphotransfer domain (DHp) containing the phosphorylatable His connected to the catalytic ATP-binding domain (CA). In hHKs, the REC-1 domain is at the C-terminal and transfers the phosphoryl group to the isolated histidine phosphotransfer protein (HPt) that forwards it to the REC-2 domain in the RR that exerts the signal. In unHKs, the REC-1 domain is connected to the HPt. In fungi, the hHK Sln1 communicates with HPt Ypd1 in order to activate RRs Skn7 or Ssk1 and the latter negatively regulates the MAP kinase cascade. A single chain of hHK and unHK is shown for simplicity reasons, while HKs naturally exist as dimers. **b** Schematic representation of domain organization of 5 hHKs from *C. thermophilum* named as hHK4, hHK5, hHK11, hHK6 (ortholog of Sln1), and hHK3 (ortholog of Nik1). The REC-1 domains of each hHK were used in the phosphotransfer experiments, as well as a construct of hHK6 (residue 691-end indicated by red dotted lines).

His-containing phosphotransfer proteins (HPt) are present in microbial signal transduction systems called phosphorelay^1,2^ which are absent in mammals. Phosphorelay is a complex version of two-component systems which are formed by a sensor protein histidine kinase (HK), with a phosphorylatable His in its dimerization domain, and an effector protein response regulator (RR), with a phosphorylatable Asp in its receiver domain (REC)^3^. While HK and RR communicate by a His-Asp phosphotransfer, phosphorelay systems use a more complex architecture of HK to communicate in three phosphotransfer steps (Fig. 1a). If the HK incorporates a REC domain (REC-1) is denoted as hybrid histidine kinase (hHK), and the first His-Asp phosphotransfer event occurs in the same polypeptide chain. Then, HPt protein containing a phosphorylatable His shuffles the phosphoryl group from the phosphorylatable Asp in the REC-1 domain to the phosphorylatable Asp in the REC domain of an RR (REC-2) in two more phosphotransfer steps (Fig. 1a). But, HPt can also be found fused to the REC-1 domain producing unorthodox HKs (unHK) generating two His-Asp phosphotransfer events in the same polypeptide chain before the phosphoryl group reaches the REC-2 domain of the RR^4^ (Fig. 1a).

HPt can be found in bacteria, fungi, and plants^5^. Gram-negative bacteria can contain several unHKs^6^ where the HPt domain can recognize the REC-2 at its cognate RR in a one-to-one interaction, although recent data indicate that unHK can phosphotransfer to non-cognate RR^7^. In turn, several bacterial hHKs communicate with an isolated HPt to reach the RR, making the specificity of protein-protein recognition more challenging as the signal is transduced by a many-to-one interaction^8–10^. Recent data on the REC-1 domain of the hHK CckA from *Caulobacter crescentus* indicates that this domain plays a rather passive role in phosphotransfer not inducing allosteric changes to control the output domain function or partner recognition^11^. Major pathogenic fungi lack unHKs but contain several hHKs and one HPt, in contrast to *Saccharomyces cerevisiae* which contains only one hHK named Sln1^12^ (Fig. 1a). The number of fungal hHKs varies between species, human pathogens such as *Candida albicans* has 3 hHKs, *Histoplasma capsulatum* has 4 hHKs, *Blastomyces dermatitidis* has 5 hHKs, *Cryptococcus neoformans* has 7 hHKs and *Aspergillus fumigatus* has 13 hHKs^13,14^. Bacterial and fungal hHK are decorated with several cytoplasmic sensor domains in the N-terminal region (PAS, PAC, HAMP, GAF, and PHY)^15^, however, fungi have a high abundance of those sensor domains which has resulted in an extensive classification of hHK from group I to group XIX according to the number and type of sensor domains^16,17^.

In fungi, the HPt accepts the phosphoryl group from REC-1 domains of various hHKs upstream of the signaling pathway and forwards it downstream to REC-2 domains present in at least two conserved RRs, Ssk1 and Skn7^5,18^. However, in *S. cerevisiae*, unphosphorylated Ssk1 stimulates the activity of MAPKs from the high-osmolarity glycerol mitogen-activated protein (Hog1), thus, connecting the phosphorelay system with the MAPK through negative regulation^19^ (Fig. 1a). The importance of HPt connecting upstream and downstream signals is reflected in fungal survival upon its mutation. The first fungal HPt identified was Ypd1 in *S. cerevisiae* (Sc_Ypd1; Sc stands for *S. cerevisiae* now on) which resulted essential for viability as its deletion produced a constitutive activation of the MAPK cascade that was lethal^18^. HPt has also been demonstrated to be essential for viability in *C. neoformans*^20^*, Neurospora crassa*^21^, *A. nidulans*^22^, and *A. fumigatus*^23^, however, HPt is dispensable in *Schizosaccharomyces pombe*^24^, and *C. albicans*^25^. These differences might be related to other signaling processes that can be independent of the Hog1-MAPK cascade, as recognized in *C. neoformans*^20^, and which could be integrated with the dynamic localization of Ypd1 that shuttles between the nucleus and the cytoplasm^23,25,26^. In line with the idea that HPt may show differences in signaling, a long N-terminal for the ortholog Mpr1p in *S. pombe* has been demonstrated to be involved in protein–protein interactions with the REC-2 domain of downstream RRs^27^. Also, Ypd1 from *C. neoformans* shows an extended N-terminal region that is important for structural stability, photostability, and binding of calcium ions^28^.

Along the years, the structure of Sc_Ypd1 isolated and in complex with the REC domain of Sln1 (REC_Sc_Sln1_), either in the absence or presence of phosphomimetic beryllium trifluoride (BeF_3_^−^)^29,30^, as well as in complex with the REC domain of Ssk1 has been obtained^31^, which has provided the first insights into recognition in fungal phosphorelay. Comparison between those complexes indicates that the presence of phosphomimetic induces the Y–T coupling mechanism proposed for the activation of bacterial RRs^32^ and proposes phosphoryl transfer through an associative mechanism^30^. As Sc_Ypd1 recognizes upstream just the REC-1 domain of Sln1, we wanted to understand the recognition and phosphotransfer mechanism associated with HPt in other fungi containing several hHKs, as there is a lack of direct biochemical evidence in this respect, evidencing that much remains unknown about phosphorelay systems in fungi^33^.

For that purpose, we have evaluated the phosphotransfer and interaction capacity between REC-1 domains derived from various hHKs, belonging to group III (hHK3; REC_hHK3_), group IV (hHK4; REC_hHK4_), group V (hHK5; REC_hHK5_), group VI (hHK6: REC_hHK6_) and group XI (hHK11; REC_hHK11_) (Fig. 1b), and HPt from the thermophilic fungus *C. thermophilum* (Ct_HPt). We have also evaluated phosphotransfer from the REC-1 domain of Sln1 (REC_Ca_Sln1_) to Ypd1 from *C. albicans* (Cal_Ypd1). Also, we have obtained the crystal structures of Ct_HPt alone and in complex with REC_hHK6_ bound to the phosphomimetic BeF_3_^−^, as well as the crystal structures of REC_hHK3_ and REC_Ca_Sln1._ This has allowed us to demonstrate that REC_hHK3_, REC_hHK5,_ and REC_hHK6_ exploit phosphotransfer in less than 1 min to Ct_HPt, despite their low affinity binding to Ct_HPt for complex formation, and propose that REC-1 domains prevent a Leu-Thr switch to stabilize phosphoryl groups at the active center promoting transient phosphorylation. Moreover, our structural studies with isolated Ct_HPt and Cal_Ypd1 have allowed us to provide a new perspective on the modularity of fungal HPt. In this sense, we have obtained the envelope structure of Cal_Ypd1 that shows an extended flexible loop that is not involved in the phosphotransfer activity.

## Results

### Phosphotransfer from REC-1 domains to Ct_HPt from *C. thermophilum*

**Fig. 2:**
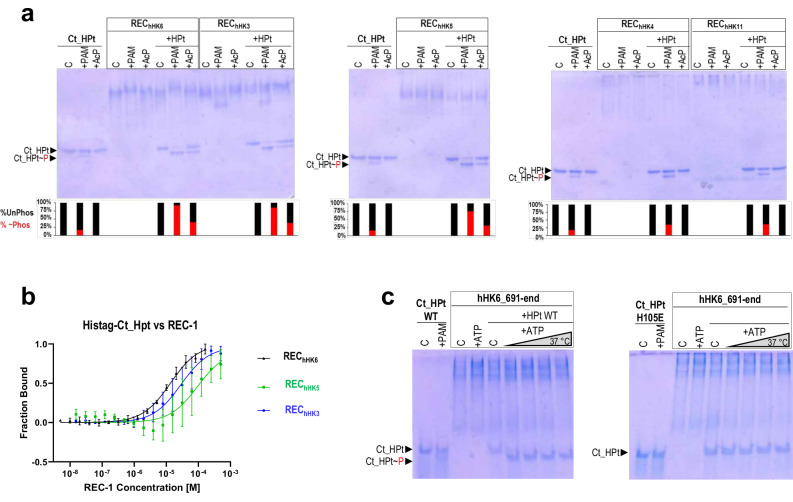
Phosphotransfer and phosphorylation from REC-1 domains from hHKs to Ct_HPt. **a** Phosphotransfer experiments from REC-1 domains of the hHKs in Fig. 1b phosphorylated first with PAM or AcP and incubated with Ct_HPt for 30 min. Control of Ct_HPt incubated with AcP or PAM for 30 min is shown. **b** Microscale thermophoresis (MST) experiments for REC_hHK6_ (black line), REC_hHK3_ (blue line), and REC_hHK5_ (green line) against labeled Ct_HPt are shown. Hyperbolic fitting (in semilog representation) of the fractional fluorescence change arising from fluorescently labeled Ct_HPt, represented as the fraction bound at different concentrations of REC-1 domains (see “Methods” for the estimation of the fraction of saturation at each concentration of the REC-1 domain). Each point is the mean for two different titrations. **c** Phosphotransfer experiments from hHK6 (fragment 691-end) phosphorylated with 5 mM ATP and incubated with Ct_HPt during 1 min, 5 min, 15 min, and 30 min. Control of Ct_HPt incubated with PAM for 30 min is shown as well. The lines containing “C” refer to the proteins without phosphodonor.

To understand if Ct_HPt showed distinct selectivity to accept phosphoryl groups from various hHKs, we produced the isolated REC-1 domains from 5 hHKs of *C. thermophilum* (Fig. 1b). Specifically, CTHT_0053860 of group III ortholog of Nik1 (hHK3), CTHT_0014080 of group IV (hHK4), CTHT_0002680 of group V (hHK5), CTHT_0050920 of group VI ortholog of Sln1 (hHK6), and CTHT_0073540 of group XI (hHK11). We studied if the isolated REC-1 domains of these hHKs, REC_hHK3_, REC_hHK4_, REC_hHK5_, REC_hHK6_, and REC_hHK11_ could transfer phosphoryl groups to the same extent to Ct_HPt. To phosphorylate the REC-1 domains, we used two well-known phosphodonors, acetyl phosphate (AcP) and phosphoramidate (PAM), the latter synthesized by our group as described^34^. Upon phosphorylation of REC-1 domains during 30 min at 37 °C, the Ct_HPt was added and native gel electrophoresis was conducted (Fig. 2a). We observed an electrophoretic mobility change for Ct_HPt phosphorylated (Ct_HPt~P) upon incubation with the REC-1 domains in the presence of either phosphodonors but in a different extent. Incubation of REC_hHK6_, REC_hHK3_, and REC_hHK5_ with PAM resulted in a maximum amount of Ct_HPt~P (quantified as ~90% for REC_hHK6_, ~85% for REC_hHK3_, and ~75% for REC_hHK5_) but when incubated with AcP the REC_hHK6,_ REC_hHK3_, and REC_hHK5_ produced less amount of Ct_HPt~P (quantified as ~40% for REC_hHK6_, ~38% for REC_hHK3_, and ~30% for REC_hHK5_). Meanwhile, REC_hHK4_ and REC _hHK11_ produced a lower amount of Ct_HPt~P (quantified as ~35%) upon incubation with PAM and no phosphorylated Ct_HPt upon incubation with AcP (Fig. 2a). Surprisingly, we observed ~15% Ct_HPt~P in the control sample of Ct_HPt incubated with PAM, thus, this indicated that Ct_HPt had a certain capacity to accept phosphoryl groups directly from PAM. This fact could account for a small amount of Ct_HPt~P upon incubation with REC_hHK6_, REC_hHK3_, and REC_hHK5_, but it corresponded to almost half the Ct_HPt~P upon incubation with REC_hHK4_ and REC_hHK11_ (~35%) indicating very low or absent phosphotransfer capacity from these two REC-1 domains (Fig. 2a). We ascribed the very low or absent phosphotransfer capacity of REC_hHK4_ and REC_hHK11_ to their low structural stability (they had a tendency to precipitate) and less capacity to interact with Ct_HPt as they showed diffuse bands in the gel, despite using the same amounts of REC-1 domains in our assay (Supplementary Fig. 1a). We conducted circular dichroism (CD) with the studied REC-1 domains observing differences in the CD spectra for REC_hHK4_ and REC_hHK11,_ with respect to the rest of REC-1 domains, which could account for structural differences (Supplementary Fig. 1b).

We also conducted phosphotransfer experiments with the REC-1 domains phosphorylated with PAM but incubated with Ct_HPt at short times (0.5 min, 1 min, and 5 min) and at room temperature (RT). Again, we observed a high amount of Ct_HPt~P after phosphotransfer from REC_hHK6_, REC_hHK3,_ and REC_hHK5_ (quantified as ~95% for REC_hHK6_ and REC_hHK3_ and ~85% for REC_hHK5_) but very low amount for REC_hHK4_ and REC_hHK11_ (Supplementary Fig. 2). To assess that phosphotransfer from REC-1 domains was being carried out on the phosphorylatable H105 of Ct_HPt, we generated the mutant H105E and conducted phosphotransfer experiments with REC_hHK6_ and REC_hHK3_ in the presence of PAM. The absence of electrophoretic mobility shift for the Ct_HPt mutant H105E in the presence of PAM, compared to WT, alone or upon incubation with the REC-1 domains indicated the absence of phosphotransfer and confirmed H105 as the only nucleophilic residue to become phosphorylated (Supplementary Fig. 3a). Also, we tested that phosphotransfer was specific for REC-1 domains as the *Salmonella* RR RcsB phosphorylated with PAM did not phosphotransfer to Ct_HPt compared to REC_hHK6_ (Supplementary Fig. 3b).

We also performed phosphotransfer from REC_hHK6_, REC_hHK3_ and REC_hHK5_ to Ct_HPt by phosphorylating the REC-1 domains using radioactive AcP (Supplementary Fig. 3c). Although we observed fainted bands ascribed to Ct_HPt~P after 15 min and 45 min of incubation, phosphorylated bands for the REC-1 domains alone, previously to phosphotransfer, were not observed, possibly indicating transient phosphorylation. In line with this fact, phosphorylation of REC-1 domains was not easily ascribed in the native gels, although a slight change in mobility could be observed for REC_hHK6_ and REC_hHK3_ in the presence of PAM (Fig. 2a and Supplementary Fig. 2).

Interestingly, we did not observe bands corresponding to complex formation between REC-1 domains and Ct_HPt which seemed indicative of a transient complex formation. Thus, we measured quantitatively the binding for complex formation using microscale thermophoresis (MST) to obtain equilibrium dissociation constants (*K*_*D*_) that could evaluate the affinity for the interactions. For that purpose, fluorescent-labeled Ct_HPt was mixed with increasing concentrations of each REC-1 domain. The binding model was fitted to 1:1 interaction and the *K*_*D*_ values obtained were in the µM range being 13 ± 0.04 μM for REC_hHK6_, 28 ± 0.1 μM for REC_hHK3_, and 94 ± 0.15 μM for REC_hHK5_. These *K*_*D*_ values indicated that the affinity for the interaction was rather weak, decreasing even more for REC_hHK5_ (Fig. 2b). Thus, REC_hHK6_ showed higher affinity binding and phosphotransfer capacity, followed by REC_hHK3_ and REC_hHK5._

Finally, to confirm that Ct_HPt phosphorylation could be obtained from a hHK, we produced recombinantly in *E. coli* a fragment of hHK6 containing the complete catalytic portion that expands from residue 691 to 1290 containing DHp-CA-REC domain (hHK6_691-end, Fig. 1b) and checked phosphotransfer to Ct_HPt WT and mutant H105E (Fig. 2c). Upon phosphorylation of hHK6_691-end with ATP and further incubation with Ct_HPt, we observed the electrophoretic mobility shift ascribed to Ct_HPt~P in the first minute of incubation, but we did not observe electrophoretic mobility shift for the mutant H105E. This fact demonstrated that phosphotransfer was conducted to the phosphorylatable H105 in Ct_HPt either from hHKs or their isolated REC-1 domains.

### Recognition of REC-1 domains to interact with Ct_HPt

**Fig. 3:**
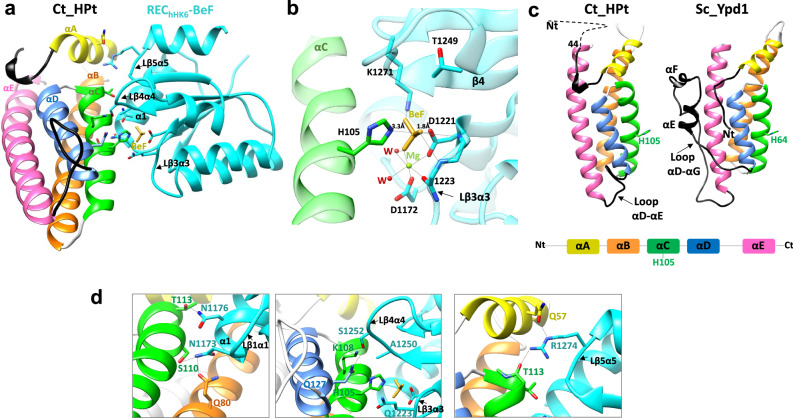
Structural studies of Ct_HPt in complex with REC_hHK6_. **a** Complex structure of REC_hHK6_ bound to Ct_HPt showing the phosphorylatable residues (H105 from Ct_HPt and D1221), the phosphomimetic BeF_3_^−^ (BeF), and residues involved in interactions from each molecule. Secondary structural elements are also labeled. **b** Zoom in on the active center for the complex REC_hHK6_-BeF:Ct_HPt highlighting the residues and waters involved in the coordination of the phosphomimetic and the Mg^2+^ ion. **c** Structural superposition of Ct_HPt with Sc_Ypd1 structure, but represented separately. Each helix α (from A to E) has been colored as depicted in the scheme. The side chain of phosphorylatable His, H105 for Ct_HPt and H64 for Sc_Ypd1 are shown. The N-terminal (the first 43 residues are disordered and absent in the Ct_HPt structure) and the loop connecting helices αD-αE in Ct_HPt and αD-αG in Sc_Ypd1 are shown in black. **d** Zoom in for the interacting areas between REC_hHK6_ and Ct_HPt that involve loop β1–α1 (Lβ1α1) and α1 (left panel), loop β4–α4 (Lβ4α4) and loop β3–α3 (Lβ3α3) (middle panel) and loop β5–α5 (Lβ5α5) (right panel) that interact with several α-helices from Ct_HPt (αA, αB, αC, and αD).

**Fig. 4:**
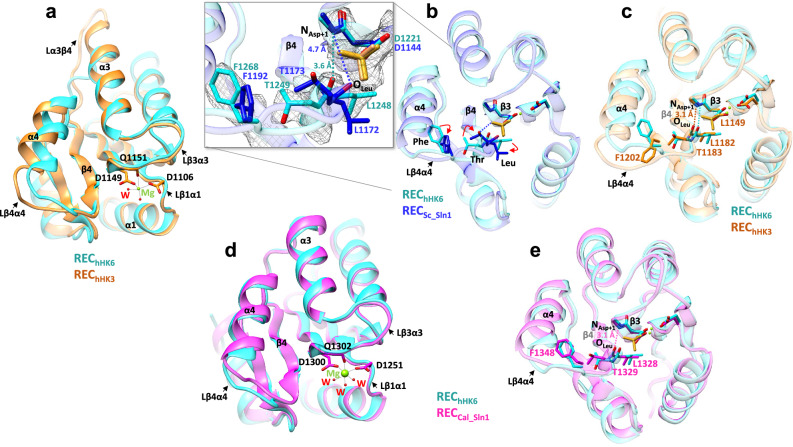
Structural insights of REC-1 domains and the Leu-Thr switch. **a** Superposed structures of REC_hHK6_ and REC_hHK3_ showing the active center for the latter and coordination of the Mg^2+^ ion with the phosphorylatable D1149, D1106, main chain oxygen of Q1151, and two water molecules. Differences in the length of α3 and loop α3–β4 (Lα3β4) between the two REC-1 domains, as well as the conformational differences between loop β4–α4 (Lβ4α4) and α4 are shown. **b** Superposition of REC_Sc_Sln1_-BeF (in dark blue) from the complex of REC_Sc_Sln1_-BeF:Sc_Ypd1 (PDB: 2R25) with REC_hHK6_-BeF (in light blue) from the complex REC_hHK6_-BeF:Ct_HPt. Residues F1192 in β5, T1173, and L1172 in β4 for REC_Sc_Sln1_-BeF involved in the Y–T mechanism and Leu-Thr switch are shown in dark blue together with the hydrogen bond between T1173 and BeF. In light blue is shown F1268 in β5, T1249, and L1248 in β4 for REC_hHK6_-BeF. In a zoomed view, the superposed active sites with electron density for REC_hHK6_. The distance between the main chain oxygen of Leu in β4 (O_Leu_) with the main chain nitrogen of N_Asp+1_ residue (distance O_Leu_-N_Asp+1_ for REC_hHK6_ is 3.6 Å and for REC_Sc_Sln1_ is 4.7 Å) is labeled. **c** Superposed structures in (**a**), REC_hHK6_-BeF (in light blue) and REC_hHK3_ (in orange) show the orientation of F1202 in β5, T1183 and L1182 in β4 for the absence of Leu-Thr switch and the distance O_Leu_-N_Asp+1_ for REC_hHK3_ is labeled (3.1 Å, in orange). **d** Superposed structure of REC_Cal_Sln1_ (in magenta) with REC_hHK6_ (in light blue) highlighting the active center for the former and coordination of the Mg^2+^ ion with the phosphorylatable D1300, D1251, main chain oxygen of Q1302 and three water molecules. Conformational differences between α4 and loop β4–α4 (Lβ4α4) are shown. **e** Superposed structures in (**d**) show the orientation of F1348 in β5, T1329, and L1328 in β4 for the absence of Leu-Thr switch, and the distance O_Leu_-N_Asp+1_ for REC_Cal_Sln1_ is labeled (3.1 Å, in magenta). Distances in all panels are shown as dotted lines.

Our phosphotransfer studies have demonstrated that REC_hHK3_, REC_hHK6_, and REC_hHK5_ can phosphotransfer in less than 1 min to Ct_HPt and that the affinity for the interaction is low, albeit with differences. Thus, to study the recognition mechanism from many-to-one at the molecular level we performed crystallization trials with these REC-1 domains alone and in the presence of Ct_HPt. We obtained crystals for REC_hHK6_ in complex with Ct_HPt and for REC_hHK3_ alone, both in the presence of phosphomimetic BeF_3_^−^ (Table 1). Surprisingly, the phosphomimetic was found bound just in the active center of the complex together with a Mg^2+^ ion (Fig. 3) while REC_hHK3_ contained just a Mg^2+^ ion bound in the active center (Fig. 4a).

**Table 1:** Data collection and refinement statistics for the obtained structures

	Ct_HPt	REC_hHK6_-BeF:Ct_HPt	REC_hHK3_	REC_Cal_Sln1_
Data collection				
Space group	C 1 2 1	P 2_1_ 2_1_ 2_1_	F 4 3 2	P 2 2_1_ 2_1_
Cell dimensions				
* a, b, c* (Ȧ)	95.88 149.33	57.25 57.47	154.27 154.27	65.16 96.27
	65.37	68.97	154.27	97.28
α, β, γ (°)	90.00 101.02	90.00 90.00	90.00 90.00	90.00 90.00
	90.00	90.00	90.00	90.00
Resolution (Ȧ)	74.67–2.40 (2.49–2.40)	68.97–2.40 (2.49–2.40)	89.07–1.90 (1.94–1.90)	97.28–1.50 (1.53–1.50)
No. reflections	236,637 (22,834)	66,052 (7181)	275,556 (17418)	723,181 (35,636)
* R*_sym_ or *R*_merge_	0.087 (0.531)	0.149 (0.926)	0.088 (1.220)	0.053 (0.922)
* R*_pim_	0.055 (0.358)	0.064 (0.388)	0.026 (0.374)	0.031 (0.532)
I/σI	10.6 (2.70)	9.2 (2.7)	22.3 (3.0)	17.3 (2.2)
Completeness (%)	99.90 (99.70)	100.0 (100.0)	100.0 (100.0)	100 (100)
Redundancy	3.39 (1.31)	7.0 (7.4)	21.3 (21.7)	7.3 (7.4)
Refinement				
* R*_work_/*R*_free_	0.24/0.27	0.22/0.27	0.21/0.22	0.19/0.21
No. atoms				
Protein	4013	1870	931	4002
Ligand/Ion	12	5	2	4
Water	86	19	45	482
B-factors				
Protein	53.39	46.60	36.80	20.91
Ligand/Ion	57.38	28.68	44.39	24.56
Water	51.52	37.65	38.11	30.90
R.m.s deviations				
Bond lengths (Ȧ)	0.004	0.002	0.005	0.006
Bond angles (°)	1.28	0.76	1.15	1.36
PDB code	8PBW	8PDC	8PHN	8PHX

Values in parentheses are for the highest-resolution shell.

The structure of the complex REC_hHK6_-BeF_3_^−^:Ct_HPt comprised one molecule of each protein in the asymmetric unit (AU) and had an interface area of 835 Å^2^. The REC_hHK6_ showed the β_5_α_5_ organization typical of REC domains connected by five loops β–α (Fig. 3a). However, loop β4–α4 seemed highly mobile with scarce electron density and side chains could not be traced. The phosphomimetic BeF_3_^−^ was stabilized in the active center by interactions with the phosphorylatable D1221 (distance between Be and O atoms is 1.76 Å), residue K1271, a water molecule, and a Mg^2+^ ion, but surprisingly, it was not bound to the conserved T1249 at the end of β4. Also, the phosphomimetic maintained a distance of 3.3 Å (between Be and N3 atoms) from the phosphorylatable H105 (Fig. 3b). Meanwhile, the Mg^2+^ ion was stabilized by additional interactions with the phosphorylatable D1221, residue D1172 in loop β1–α1, the main chain carbonyl oxygen of Q1223 and two water molecules (Fig. 3b). Superposition of the complex structure REC_hHK6_-BeF:Ct_HPt with REC_Sc_Sln1_-BeF:Sc_Ypd1 (PDB: 2R25) showed a similar spatial orientation and similar distance Be–N3 (Supplementary Fig. 4a), but differences in conformation were observed for the loop β4–α4 and position of α4.

Meanwhile, the Ct_HPt structure, following the nomenclature of Sc_Ypd1, consisted of a bundle of helices formed by five helices named αA (residues 52–61), αB (residues 70–93), αC (residues 97–114), αD (residues 117–131), and αE (comprises 143–172), locating the phosphorylatable H105 in αC (Fig. 3a, c). Interestingly, αE in Ct_HPt corresponded to αG in Sc_Ypd1, thus, the loop αD–αE in Ct_HPt corresponded to the loop αD–αG in Sc_Ypd1 as it contains two small helices αE and αF (Fig. 3c). We checked the length of this loop αD–αE in other fungal species and observed a similar length in the human pathogens *A. fumigatus*, *H. capsulatum*, *B. dermatitidis*, *C. neoformans*, and *C. auris*, albeit in *C. albicans* which was even longer than in *S. cerevisiae* (Supplementary Fig. 4b). Also, in the Ct_HPt structure, the first ~43 residues of the N-terminal region were disordered as could not be traced due to absence of electron density (Fig. 3c). Interestingly, HPt of previously mentioned human pathogens, except *Candida*, also show long N-terminal extensions with predicted disordered structures inferred from modeled structures obtained with AlphaFold^35,36^ and Robetta^37^ servers (Supplementary Fig. 5).

In the complex, residues of REC_hHK6_ involved in interactions with Ct_HPt were located in loop β1–α1 (D1172 and N1173), α1 (T1174, V1175, N1176, V1179 and R1182), loop β3–α3 (Q1223), loop β4–α4 (A1250 and S1252), and loop β5–α5 (P1272 and R1274) (Fig. 3a, d and Supplementary Table 1). In Ct_HPt, the residues involved in interactions with REC_hHK6_ corresponded to residues in αA, αB, αC, and αD (Supplementary Table 1). The majority of contacts involved α1 and loop β1–α1 in REC_hHK6_ interacting with αB and αC in Ct_HPt that were supported by peripheral contacts between loop β3–α3 and loop β4–α4 interacting with αC and αD, as well as between loop β5–α5 interacting with αA and αC (Fig. 3d).

We obtained the structure of REC_hHK3_, which contained one molecule in the AU, and we superposed it into REC_hHK6_. Both structures demonstrated overall structural similarity (rmsd value of 1.4 Å for 116 residues aligned), although, α3 and loop α3–β4 were longer in REC_hHK3_ (Fig. 4a). In REC_hHK3_ the Mg^2+^ ion was stabilized in the active center by interactions with the phosphorylatable D1149, residue D1106 in loop β1–α1, the main chain carbonyl oxygen of Q1151 in loop β3–α3 and two water molecules (Fig. 4a). Also, the conformation of loop β4–α4 seemed highly mobile with scarce electron density, as observed in REC_hHK6_, and the conformation between both REC-1 domains was different affecting to the spatial position of α4 (Fig. 4a). Superposed REC_hHK3_ in the complex revealed close contacts with Ct_HPt just for two residues located in α1 which had flexible long side chains (R1112 and K1161); thus, recognition of REC-1 domains seems to involve subtle conformational changes.

We also compared the conservation of interacting residues in REC_hHK6_ with REC_hHK3_, REC_hHK5_, REC_hHK4_, and REC_hHK11_, and with REC_Sc_Sln1._ For REC_hHK4_, REC_hHK5_, and REC_hHK11_, we have modeled their structure with AlphaFold^35^ to locate the interacting residues observed in the complex (Supplementary Fig. 6). The majority of them were conserved in the REC-1 domains except for T1174 (variable in all REC domains), Q1223 (His in REC_hHK5_ but Glu in REC_hHK4_ and REC_hHK11_) and S1252 (Ala in all RECs except Val in REC_hHK5_) (see Supplementary Table 2 and Supplementary Fig. 6). There were two conserved Asn residues at loop β1–α1 and α1 in all REC-1 domains except in REC_hHK11_ which showed a disordered N-terminal lacking those secondary structural elements, as can be observed in its modeled structure (Supplementary Table 2 and Supplementary Fig. 6). Absence of the Asn residues in REC_hHK11_ could explain in part lack of phosphotransfer to Ct_HPt. Due to the high conservation of the Asn residues in loop β1–α1 and α1, we assessed their impact on recognition and catalysis. We produced single and double mutants for REC_hHK6_ (N1173A and N1173A/N1176A) and REC_hHK3_ (N1107A and N1107A/N1110A) and conducted phosphotransfer experiments to Ct_HPt. Interestingly, these mutants could phosphotransfer similarly as fast as the WT, within the first minute (Supplementary Fig. 7a). Thus, just a reduction of four-fold in the protein ratio of REC_hHK6_ vs Ct_HPt allowed to observe a reduction in the amount of phosphorylated-Ct_HPt, that was more evident for the double mutant (~80% phosphorylated in WT and ~40% phosphorylated for double mutant) (Supplementary Fig. 7b). Meanwhile for REC_hHK3_ both the WT and double mutant showed a similar reduction in phosphorylated Ct_HPt (~40% phosphorylated). We also conducted MST to assess the equilibrium dissociation constant between the double mutant REC_hHK3_ (N1107A/N1110A) with Ct_HPt. The analysis resulted in a *K*_*D*_ of 46 ± 0.07 μM, demonstrating an affinity of a similar range for the WT, albeit a bit lower (Supplementary Fig. 7c). Thus, decreasing the ratio of REC-1 domain has allowed confirming higher phosphotransfer capacity of REC_hHK6_ vs REC_hHK3_ and that the absence of the conserved Asn residues had lower effect in the phosphotransfer, probably due to a compensatory effect of the other interacting areas.

Finally, we observed that residues of Ct_HPt involved in interactions with REC_hHK6_ were rather conserved in Sc_Ypd1 and other HPt from human pathogens such as *C. albicans*, *C. auris*, *A. fumigatus*, *H. capsulatum*, *B. dermatitidis* or the plant pathogen *N. crassa* (Supplementary Table 3 and Supplementary Fig. 8). In this way, ~82% of interacting residues located in αB and αC are conserved while ~75% of total interacting residues are conserved as well. Thus, high conservation for the interacting residues in HPt and REC-1 domains may indicate reduced residue coevolution between fungal species, as well as maintenance of reduced specificity for recognition.

### Absence of a Leu-Thr switch in β4 of REC-1 in *C. thermophilum* and *C. albicans*

According to the Y–T mechanism initially described for CheY^38,39^ and observed in many other RRs^32^, binding of the phosphomimetic in the REC domain induces the reorientation of the conserved Thr/Ser in β4 and Tyr/Phe in β5 towards the active center. In the complex REC_hHK6_-BeF:Ct_HPt, the conserved Thr in β4 and the Phe in β5 do not reorient towards the active center when the phosphomimetic is bound, thus, the conserved Thr (T1249) is not bound to the phosphomimetic. This contrasts with the configuration observed in the complex REC_Sc_Sln1_-BeF:Sc_Ypd1 (PDB: 2R25) where the phosphomimetic interacts with the hydroxyl group of the conserved Thr and reorients both residues towards the active center (Fig. 4b).

Meanwhile, a closer inspection of the active center for REC_hHK3_ allowed us to observe that the C-terminal of the highly conserved β4, containing the motif LTA comprised by the conserved Thr (T1183) (Supplementary Fig. 9), formed a hydrogen bond (distance ~ 3 Å) with the C-terminal of β3 (Fig. 4c). Specifically, this bond involved the main chain carbonyl oxygen (O) of Leu in β4 (L1182) and the main chain nitrogen (N) of the residue after the phosphorylatable D1149 (Asp + 1) in β3 (O_Leu_-N_Asp+1_ distance). When we compared this configuration in the active center of REC_Sc_Sln1_-BeF (PDB:2R25), the accommodation of the phosphomimetic broke this hydrogen bond (distance of ~4.7 Å) and Leu in β4 (L1172) changed its side chain rotamer moving χ_1_ angle from −50° to −180° (Fig. 4b). This change allows the stabilization of the Leu in a hydrophobic pocket and pulls β4 to reorient the conserved Thr (T1173) towards the active center to interact with the phosphomimetic (Fig. 4b). Then, following the Y–T mechanism, movement of the Thr is transmitted to loop β4–α4 and α4 inducing the reorientation of Phe (F1192) in β5 towards the active center. Interestingly, in REC_hHK6_-BeF, the O_Leu_-N_Asp+1_ distance between Leu in β4 (L1248) and phosphorylatable D + 1 in β3 is much shorter (~3.6 Å) and Leu had not changed the rotamer impairing the Leu-Thr switch and the reorientation of conserved Thr in β4 (T1249) and Phe in β5 (F1268) towards the active center (Fig. 4b).

We believe that the lack of the Leu-Thr switch may be behind the transient phosphorylation at the active center, a feature that can be integrated with effective phosphotransfer from REC_hHK6_, REC_hHK3_, and REC_hHK5_ when the selectivity for Ct_HPt recognition is reduced. We propose that this functionality may explain how several REC-1 domains from hHKs can phosphotransfer to the same one HPt.

Motivated by our findings and to analyze the Leu-Thr switch in another fungus, we set up crystallization trials with the REC-1 domain of *C. albicans* (REC_Cal_Sln1_) in the presence of phosphomimetic, either isolated or together with Cal_Ypd1. We obtained crystals for REC_Cal_Sln1_ isolated which contained four molecules in the AU (Table 1 and Supplementary Fig. 10a). Again, the molecules did not contain phosphomimetic in the active center, but contained a Mg^2+^ ion bound stabilized by interactions with the phosphorylatable D1300, residue D1251 in loop β1–α1, main chain carbonyl oxygen of Q1302 in loop β3–α3 and three water molecules (Fig. 4d). Also, the overall structure was similar to REC_hHK6_ (rmsd of 0.7 Å in 115 residues), but, the loop β4–α4 was ordered and well defined although its conformation slightly differed from REC_hHK6_, as well as the position of α4 (Fig. 4d, e).

The structure of REC_Cal_Sln1_ also provided two interesting features. On one side, the N-terminal H1243 in one molecule was located at a close distance (<6–8 Å) of the phosphorylatable D1300 in another neighbor molecule, however, the distance was not close enough to stabilize the phosphomimetic as it does a catalytic His (Supplementary Fig. 10b). On another side, we observed that a Cys in α4 (C1340), which is conserved in the REC-1 domains studied (Supplementary Fig. 9), was connected to a large electron density that may account for a posttranslational modification that we could not identify unambiguously, despite the high resolution of the electron density map (1.5 Å).

**Fig. 5:**
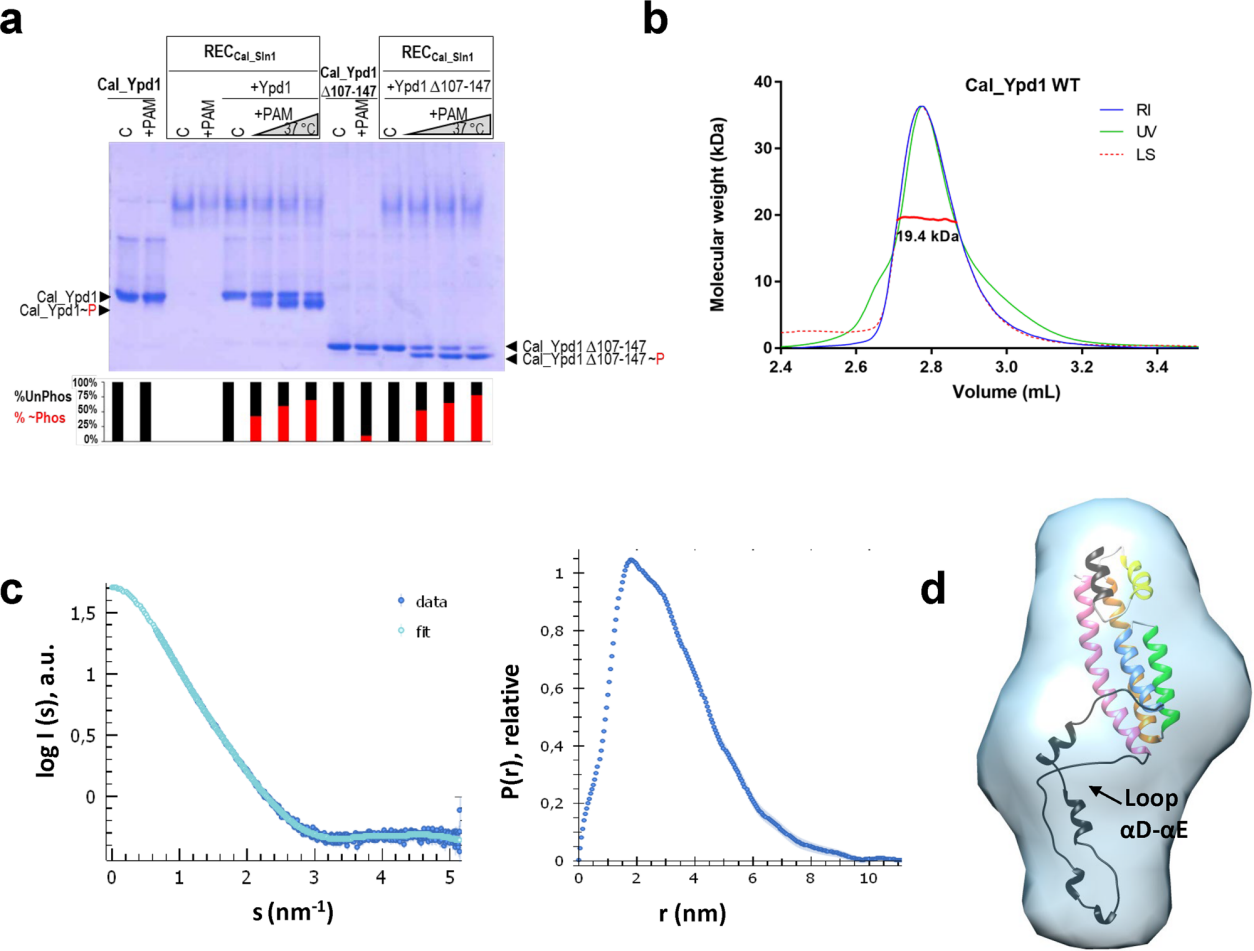
Functional and structural analysis of Cal_Ypd1. **a** Phosphotransfer experiments of REC_Cal_Sln1_ phosphorylated with 50 mM PAM for 30 min to Cal_Ypd1 variants WT and Δ107–147 (deletion of loop αD–αE) incubated during 1 min, 5 min, and 15 min. Controls of Cal_Ypd1 variants in the absence and presence of PAM have been added. The mobility shift of phosphorylated Cal_Ypd1 variants, compared to non-phosphorylated, upon phosphotransfer is observed. The lines containing “C” refer to the proteins without phosphodonor. **b** SEC-MALS data obtained with Cal_Ypd1 WT. Absorption of UV light (in green), refractive index (RI, in blue), and light scattering (in dashed red) are traced in the chromatograms. The calculated MW is represented in red with the value written below. **c** SEC-SAXS data obtained for Cal_Ypd1 WT. The left panel shows the scattering curve with experimental data in dark blue and the fitted curve in light blue. The right panel shows the pair-wise distance distribution function indicating a *D*_max_ of 115 Å. **d** Envelope structure of Cal_Ypd1 WT obtained from DAMAVER containing a model structure of Cal_Ypd1, obtained from the Robetta server, using SASREF. The secondary structure is colored as indicated for Ct_HPt in Fig. 3c. The N-terminal and the long-extended loop αD–αE are colored in black.

We also analyzed the active center of REC_Cal_Sln1_ and, as expected, in the absence of phosphomimetic, the O_Leu_-N_Asp+1_ distance between Leu in β4 (L1328) and Asp + 1 in β3 corresponds to a hydrogen bond (~3 Å) (Fig. 4e). In this way, all four molecules of REC_Cal_Sln1_ lack the Leu-Thr switch so Thr in β4 (T1329) and Phe in β5 (F1348) were oriented away from the active center (Supplementary Fig. 10b). Since REC_Cal_Sln1_ showed an inactive state in the crystal, we tested if it was functional, thus, we conducted phosphotransfer experiments to Cal_Ypd1_._ For that purpose, we first phosphorylated REC_Cal_Sln1_ with PAM and then incubated it with Cal_Ypd1 WT (Fig. 5a). In the native gel, upon incubation of both proteins, a new band ascribed to phosphorylated Cal_Ypd1 was observed which increased over time from 40% to 70% of phosphorylated molecules in the interval of 1 min to 15 min of incubation, respectively, indicating the functionality of REC_Cal_Sln1._

The fact that REC_hHK6,_ REC_hHK3_, and REC_Cal_Sln1_ were functional but showed inactive states, led us to analyze the importance of the conserved Thr in phosphotransfer. We produced mutants to Ala at each REC-1 domain, T1249A-REC_hHK6_, T1183A-REC_hHK3_, and T1329A-REC_Cal_sln1_, then, phosphorylated them with PAM and conducted phosphotransfer experiments (Supplementary Fig. 11). In contrast to the WT proteins, T1249A-REC_hHK6_ did not produce phosphorylated Ct_HPt and T1183A-REC_hHK3_ produced just ~50% of phosphorylated Ct_HPt. Meanwhile, T1329A-REC_Cal_sln1_ could produce phosphorylated Cal_Ypd1 as WT. This data indicated that the Thr was important in phosphotransfer, probably to stabilize the phosphoryl group accepted from phosphodonor and during phosphoryl transfer to Ct_HPt, albeit to a different extent at each REC-1 domain.

### Structural characterization of Cal_Ypd1 by SEC-SAXS

To dissect the conformation of the long loop αD–αE, we conducted a structural analysis of Cal_Ypd1. Size exclusion chromatography coupled to multiangle light scattering (SEC-MALS) indicated it was monomeric in solution as judged by the calculated molecular weight (MW) (19.4 kDa; theoretical MW is 19.3 kDa) (Fig. 5b). As we could not obtain the crystal structure of Cal_Ypd1, we conducted SEC coupled to small angle X-ray scattering (SEC-SAXS) that allowed us to obtain the envelope structure of Cal_Ypd1 in solution. The SAXS data demonstrated no protein aggregation in the sample and the Guinier plot showed that the radius of gyration (Rg) of Cal_Ypd1 was 25.4 Å with an estimated MW of 14.7 kDa and a *D*_max_ of 115 Å suggesting an extended conformation (Fig. 5c). Indeed, the Kratky plot indicated the presence of a flexible region (Supplementary Fig. 12a). An envelope model was constructed from the experimental scattering profiles using DAMMIF^40^ and we used a model structure obtained from the Robetta server^37^ to dock it in the envelope using rigid body refinement with SASREF (Fig. 5d). The model structures of Cal_Ypd1 indicated that the long loop αD–αE had to be extended to fit in the SAXS envelope as a monomeric species, in contrast to loop αD–αG in Sc_Ypd1 that is constrained towards αE (Fig. 3c).

To test if the long loop αD–αE had any role in phosphotransfer, we produced a mutant variant of Cal_Ypd1 lacking residues 107–147 of the long loop αD–αE (∆107–147). Then, we phosphorylated REC_Cal_Sln1_ with PAM and incubated it with the mutant Cal_Ypd1 (Fig. 5a). As shown in the native gel, the mutant ∆107–147 changed the electrophoretic mobility at the first minute of incubation with phosphorylated REC_Cal_Sln1_ indicating that the long loop αD–αE was not interfering in the reaction. Similarly to Ct_HPt, incubation of Cal_Ypd1 ∆107–147 with PAM produced a small amount of phosphorylated protein, a phenomenon less pronounced in the WT, ensuring the functionality of the produced variants.

Finally, we checked the length of the loop αD–αE in other *Candida* species, since *C. auris* showed a short loop αD–αE, and observed that *C. tropicalis* and *C. parapsilosis* showed even larger lengths, being larger for the latter (Supplementary Fig. 13). Thus, the length of this long loop could be related to unknown additional functions.

### Structure of isolated Ct_HPt

**Fig. 6:**
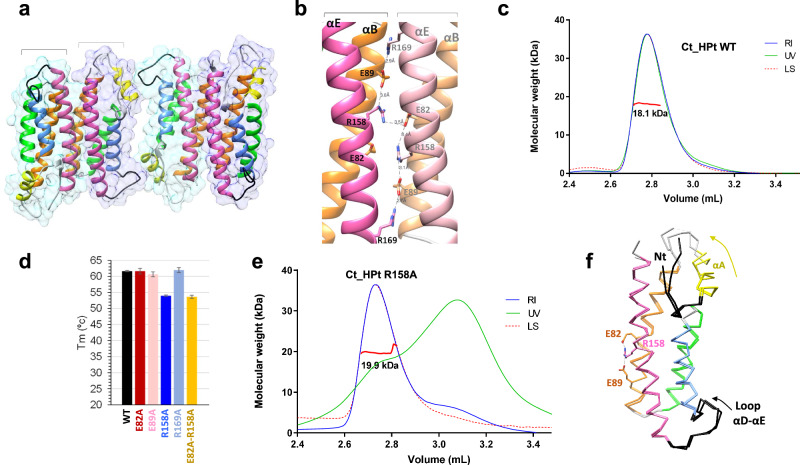
Structural and functional analysis of Ct_HPt. **a** AU composition for the crystal structure of Ct_HPt alone comprised of four molecules. The secondary structure is colored as indicated in Fig. 3c. **b** Identification of salt bridge interactions in the interface between two molecules of Ct_HPt involving the same residues in αB and αE. Residue R158 is involved in inter-chain contacts with E82 and intra-chain contacts with E82 and E89 while R169 is involved in inter-chain contact with E89. **c** SEC-MALS data obtained with Ct_HPt WT. Absorption of UV light (in green), refractive index (RI, in blue), and light scattering (in dashed red) are traced in the chromatograms. The calculated MW is represented in red with the value written below. **d** Table representing the Tm values obtained for the WT and mutant variants involved in salt bridge formation in the crystal. **e** SEC-MALS data obtained with Ct_HPt R158A. Absorption of UV light (in green) has been normalized and refractive index (RI, in blue), as well as light scattering (in dashed red), are traced in the chromatograms. The calculated MW is represented in red with the value written below. **f** Structural comparison of Ct_HPt alone and in the complex showing slight conformational changes in the N-terminal, αA, loop αA–αB, and long loop αD–αE. The intra-chain salt bridge E82-R158-E89 is observed in the complex structure as well.

To analyze differences in the structure of Ct_HPt before and after binding to REC_hHK6_, we obtained the crystal structure of isolated Ct_HPt (Fig. 6a and Table 1). It contained four molecules in the AU and each molecule contributed to an interface area of ~800 Å^2^ between two molecules (Fig. 6a). The interface area contained several salt bridges between two Arg residues located in αE (R158 and R169) and two Glu residues located in αB (E82 and E89) (Fig. 6b). Interestingly, R158 formed intrachain salt bridges with E82 and E89, as well as an interchain salt bridge with E82, while R169 formed an interchain salt bridge with E89 (Fig. 6b). According to the EPPIC and PISA servers this interface was the result of crystal packing and SEC-MALS analysis with Ct_HPt indicated a MW of 18.1 kDa (theoretical MW is 18.7 kDa) demonstrating the presence of the monomeric species in solution (Fig. 6c). However, to assess the impact on protein stability of the residues involved in salt bridge formation, we produced single mutants E82A, E89A, R158A, R169A, and a double mutant R158A/E82A. A thermal shift assay revealed changes in the thermal denaturation temperature (Tm) only for R158A and R158A/E82A which dropped to 54 °C in comparison with the Tm of 61.5 °C for the WT (Fig. 6d). Also, SEC analysis for the mutants showed a change in their elution profile in comparison with the WT, with a second peak eluting at a lower volume (Supplementary Fig. 14a). However, SEC-MALS analysis with mutant R158A identified a single monomeric species in solution with a MW of 19.9 kDa similar to WT (Fig. 6e). Thus, the salt bridges involving R158 seemed to play a relevant role in the stability of the helix bundle. We determined the structure of the mutant R158A, albeit at a low resolution of 3.4 Å (Supplementary Table 4), observing two molecules in the AU with no differences in the helix bundle with respect to the WT (rmsd ~0.6 Å for 128 residues) but in the crystal packing. The absence of R158 changed the electrostatic nature of the interface and allowed the approach of αE in both molecules to a distance of ~4 Å between Cα of A155–A155 (Supplementary Fig. 14b). Thus, differences in SEC analysis could be ascribed to differences in the hydration shell of the protein.

Interestingly, in Sc_Ypd1, the Arg residue in αE is conserved but the Glu residues in αB are Gln, thus, the salt bridge does not form. However, these ionic residues involved in the salt bridge are rather conserved in other HPt from the human fungal pathogens included in this study, the position of R158 is generally occupied by basic residues (Lys or Arg, but Glu in *B. dermatitidis*), position E82 is generally Glu (Lys in *C. auris*) and position E89 is generally acidic (Asp and Glu, and Lys in *B. dermatitidis*) (Supplementary Fig. 4b).

Finally, the structural comparison between Ct_HPt isolated and bound to REC_hHK6_ demonstrated high structural similarity between them (rmsd of ~0.9 Å for 129 residues), although slight structural changes were observed in the N-terminal region, αA, loop αA–αB and loop αD–αE (Fig. 6f). For these structural elements, just αA is involved in interactions with REC_hHK6_, specifically to the loop β5–α5, which corresponds to a peripheral area of interaction. Meanwhile, the intrachain salt bridge nucleated by R158 in αE with E82 and E89 in αB is also present and has not been disturbed (Fig. 6f). This fact indicates that Ct_HPt shows little plasticity, a useful conserved feature to be recognized similarly by several REC-1 domains.

## Discussion

Phosphorelay systems, based on phosphotransfer between His-Asp residues, are signal transduction systems widely present in bacteria but also present in fungi and plants^41,42^. These systems use phosphoryl labile residues to ensure rapid activation and response, but, for long-term activation, an extended signaling event is needed and/or a constant signal detection that ultimately avoids unspecific activation^43^. The signaling event is extended thanks to the emergence of hHKs and HPt proteins, or their combination as observed in unHKs, to create multiple phosphotransfer steps. In fungi, it is generally recognized that HPt can accept phosphoryl groups from various REC-1 domains derived from hHKs, although in *S. cerevisiae* there is just one hHK^33^. Direct biochemical evidence for phosphotransfer from various hHKs to HPt has been awaiting, but it is now demonstrated in our studies. To develop that promiscuity, REC-1 domains should show some degree of conservation in interacting areas and HPt should show a reduced interaction specificity for REC-1 domains.

The analysis of the complex structure between REC_hHK6_ bound to the bundle of helices in Ct_HPt has a lower interface area (835 Å^2^) than the interface area between the four-helix bundle of HKs (DHp domain) interacting with the REC domain as observed in the complex structure of HK853-RR468 (931 Å^2^ for PDB: 3DGE)^44^ and in the DesK–DesR complexes (940 Å^2^ for PDB: 7SSI and ~1000 Å^2^ for 7SSJ)^45^. The interacting residues of REC_hHK6_ in the complex are located in α1 and various loop areas (loop β1–α1, loop β3–α3, loop β4–α4, and loop β5–α5). Meanwhile, in RR468 and DesR the majority of interactions with DHp locate in α1 and loop β5–α5 with additional interactions for DesR in α5 and loop β4–α4. This difference is due to a more central docking position for REC_hHK6_ than for RR468 (50° apart) compared to the α1 where the phosphorylatable His sits (Supplementary Fig. 15). In this way, REC_hHK6_ uses several loops to interact with Ct_HPt reducing the contact interface area and the recognition capacity. Interestingly, our measurements on the binding kinetics for interaction resulted in *K*_*D*_ values ~ 13–90 µM. These values indicate low-affinity binding when compared to the ones observed between cognate pairs in TCS with *K*_*D*_ values of ~1–2 µM and non-cognate pairs which do not show phosphotransfer with *K*_*D*_ values > 35–75 µM^46^. The higher affinity in TCS may be derived from the increase in the shared interface area, however, *K*_*D*_ values for the complex between REC_Sc_Sln1_ and Sc_Ypd1 show similar affinity values ~ 0.6–1 µM range^47,48^. In the plant *Arabidopsis thaliana*, binding studies between the REC-1 domain of hHK AHK5 with three HPt proteins AHP1, AHP2, and AHP3 show *K*_*D*_ values ~ 2.7–4.4 µM^49^ which are slightly higher than the observed for TCS and *S. cerevisiae* phosphorelay system, but still lower than the values for the *C. thermophilum* phosphorelay systems. Thus, a reduction in the affinity for protein recognition could favor promiscuity in a many-to-one or one-to-many scenario and explain why CheY could slightly phosphotransfer to Sc_Ypd1^50^. However, a reduction in the binding affinity for recognition does not directly correlate with low specificity for phosphotransfer. The complex structure between CheA_3_ and CheY_6_ from *Rhodobacter sphaeroides* shows a reduced interface (~600 Å^2^) and low affinity for interaction (*K*_*D*_ value of 218 µM), but CheA_3_ shows specificity for phosphotransfer which could be rewired upon substitution of few residues in the α1 of several CheYs^51^. In the case of Ct_HPt, it shows a certain degree of specificity to recognize REC-1 domains, reflected by the lack of phosphotransfer between RcsB and Ct_HPt, which could be explained by an incompatible interacting interface and a tendency of RcsB to dimerize upon phosphorylation. Also, the substitution of two conserved Asn residues in α1 of REC_hHK6_ and REC_hHK3_ had a mild effect on phosphotransfer. Thus, a search to find key specific residues would allow us to understand the contribution between recognition and specificity.

But which areas are involved in providing low affinity for complex binding and recognition? In bacterial RRs, the loops β3–α3 and β4–α4 are known to show conformational changes upon phosphorylation, thus, we could envision that their conformational changes could help to promote the association or dissociation of the complex. Comparison of the REC_hHK6_ bound to Ct_HPt and REC_hHK3_ alone indicates that loop β3–α3 does not change overall, however, loop β4–α4 is quite loose and shows conformation variability affecting also the position of α4 which could reduce recognition capacity. Recent structural studies on the REC-1 domain (intermediary REC) from hHK CckA in *C. crescentus* have detected local backbone perturbations around the phosphorylation site, most prominently at the end of β4 and β4–β5 linker (where α4 is located), and absence of allosteric response upon BeF_3_^−^ binding which was ascribed to a passive role for phosphotransfer^11^. Those results are in line with the conformation variability in loop β4–α4 observed in our structural data, indicating that REC-1 domains can exploit flexibility in this area to modulate recognition capacity in order to affect the active site.

In our complex structure, we have trapped a transition state where phosphorylated REC_hHK6_ is bound to Ct_HPt just before phosphotransfer takes place, similarly as observed in the complex structure of REC_Sc_Sln1_-BeF:Sc_Ypd1^30^. However, REC_hHK6_ showed an inactive state, according to the Y–T mechanism, as the conserved Thr was not bound to the phosphomimetic. Also, the Leu-Thr switch coupled with the Phe, observed when phosphomimetic was bound at the active center of REC_Sc_Sln1_^30^ was absent. These features can explain why accommodation of phosphomimetic in REC_hHK6_ did not distort at a high degree the O_Leu_-N_Asp+1_ distance (~3.6 Å) compared with REC_Sc_Sln1_-BeF_3_^−^ (PDB: 2R25; ~4.7 Å) and REC_hHK3_ or REC_Cal_Sln1_ (~3 Å). We have analyzed the active site structure of several REC domains in the absence and presence of BeF_3_^−^ (PhoB, DrrB, DesR, RR468, ArcA, KdpE, CheY, and REC_Sc_Sln1_) to find rearrangements due to phosphomimetic binding. In the absence of phosphomimetic, the O_Leu_-N_Asp+1_ distance between Leu (or Val in CheY) in β4 and Asp + 1 in β3 is ~3 Å. However, the distance increases to ~4.5 Å in the presence of phosphomimetic (Supplementary Table 5), facilitating the interaction with the conserved Thr (or Ser in KdpE) and the main nitrogen of Thr + 1 in loop β4–α4 (distance ~3 Å) to allow its stabilization and the acquisition of the active conformation. These REC domains also show the Leu-Thr switch upon phosphomimetic binding, with the exception of CheY which has Val instead of Leu. We hypothesize that the lack of a Leu-Thr switch might serve to reduce the binding strength of the phosphomimetic in the active site to facilitate its stay and release. Indeed, the structures of HK853:RR468 and DesK:DesR in the phosphatase state show an intermediate state of the Leu rotamer and a shorter O_Leu_-N_Asp+1_ distance (Supplementary Table 5).

The absence of the Leu-Thr switch was observed in the structures of REC_hHK3_ and REC_Cal_Sln1_ which were found bound just to Mg^2+^ion, although we added phosphomimetic in our crystallization mixture. In general, binding of Mg^2+^ ion in the active center is not sufficient to induce the Y–T coupling mechanism, but in some cases, the presence of Mg^2+^ can activate similarly as the phosphomimetic, as observed in the RR ArlR from *Staphylococcus aureus*^52^. Interestingly, the inactive state is also observed in REC-1 domains from hHKs of the plant *A. thaliana*. The REC-1 domain of hHK CKI1 in the presence of BeF_3_^−^ which contains Val-Ser in β4 instead of Leu-Thr, shows a distance between O_Val_ in β4 and N_Asp+1_ of 3 Å, and the Ser is not bound to the phosphomimetic^53^. Also, the REC-1 domain of hHK AHK5 in complex with AHP1 bound to Mg^2+^ ion, which contains Met-Thr in β4, shows an O_Met_-N_Asp+1_ distance of 3.3 Å^49^.

The sequence Leu-Thr in β4 is highly conserved between bacterial RRs, representing 53% in *E. coli* RRs. However, the Leu-Thr switch can hold residue variability as other hydrophobic residues such as Val, Ile, and Met can occupy the position of Leu while Thr can be substituted with Ser, although, these combinations are present in less than 10% of RRs (ArlR contains Ile-Thr in β4)^52^. In the case of *E. coli* CheY, the best structural characterized RR, it contains Val-Thr in β4, thus, the movement of Val in the active conformation is more subtle than expected for a shorter sidechain. But as we mentioned previously, the Leu-Thr switch conservation is visible in several bacterial RRs (Supplementary Fig. 9). In fungi, the combination Leu-Thr in β4 is present in the three hHKs of *C. albicans* Sln1, Nik1, and Chk1 while in *C. thermophilum* is present in hHK6 and hHK3, as hHK5 contains Leu-Ser, hHK4 contains Val-Ser, and hHK11 contains Val-Thr (Supplementary Fig. 9). Despite the absence of Leu-Thr switch in our structures, the substitution of the conserved Thr in the REC-1 domains revealed its importance during phosphoryl transfer (accepting phosphoryl group from phosphodonor or releasing phosphoryl group to the phosphorylatable His in Ct_HPt), a reaction that could be influenced by the nature of non-conserved residues around the active site. Detailed studies conducted in CheY have studied the role of non-conserved residues in Asp + 2 (D + 2) at loop β3–α3, as well as in Thr + 1 (T + 1) and Thr + 2 (T + 2) at loop β4–α4 to influence autodephosphorylation rates compared to autophosphorylation rates. For REC_hHK6_ and REC_Cal_Sln1_ D + 2 is Gln and T + 2 is Phe, for REC_hHK3_ D + 2 is Gln and Thr + 2 is His and for REC_hHK5_ D + 2 is His and T + 2 is Asn (Supplementary Fig. 9). Combinations Gln-Phe and Gln-His in *E. coli* CheY have an autodephosphorylation rate of ~0.3 min^−1^, around 8-times slower than CheY WT (2.2 min^−^^1^) but 20-times faster than *E. coli* PhoB (0.015 min^−^^1^)^54,55^. However, the autodephosphorylation rates of CheY were inversely correlated with autophosphorylation rates suggesting differences in the transition state of both reactions. Additional experiments with CheY variants at D + 2/T + 2 mimicking Sc_Sln1, Ssk1, and Ssk7 indicate that the REC domain may have the catalyst machinery for phosphoryl transfer but HPt is essential for recognition and reaction speed^56^.

We wondered if transient phosphorylation at the active center could favor phosphotransfer reversibility. Although we did not test reversibility, we did not observe accumulation of non-phosphorylated Ct_HPt over time, even upon mixing REC-1 domain and Ct_HPt with PAM at the same time (Supplementary Fig. 16a). We determined the structure of the phosphomutant Ct_HPt H105E at 2.4 Å (Supplementary Table 4) which showed a similar structure than Ct_HPt in the complex (rmsd of 0.7 Å for 125 residues). However, the superposition of the phosphomutant to the complex showed few clashes with residues of REC_hHK6_ involved in complex interactions, thus, supporting that subtle changes in the side chain conformation of a few residues could prevent phosphotransfer (Supplementary Fig. 16b). Recent data on DesK–DesR has pointed out that reversibility seems to involve a structure-encoded allostery process where substitution Q10A in DesR can substantially increase the phosphoryl-transfer reversibility by reducing the ability to dimerize^45^. Residue Q10A corresponds to a conserved Asn located in loop β1–α1 involved in interactions at our complex and its substitution (N1173 in REC_hHK6_ and N1107 in REC_hHK3_) barely affected the phosphotransfer capacity and slightly decreased the binding affinity to Ct_HPt (Supplementary Fig. 7). Thus, we checked if the REC-1 domains could be involved in oligomerization by conducting SEC-MALS experiments (Supplementary Fig. 17). REC_hHK6_ run as monomer and dimer, albeit the latter in less proportion which shifted at some degree to monomer upon addition of BeF_3_^−^ and almost disappeared with PAM. Further incubation of REC_hHK6_ with Ct_HPt did not show complex formation in the absence and presence of phosphomimetic (Ct_HPt was not affected by BeF_3_^−^ or PAM). Meanwhile, REC_hHK3_ showed a monomeric species and a small amount of oligomer that we could assign to a trimer, detected in the crystal by the PISA server (buried area of 3935.6 Å^2^ and a ΔG^diss^ of 38.9 kcal/mol indicating stability), which shifted to monomer in the presence of PAM (Supplementary Fig. 17). REC_hHK5_ remained as a monomer (Supplementary Fig. 17). Interestingly, according to the PISA server REC_Cal_Sln1_ also formed two dimers in the crystal (buried area of ~1800 Å^2^ and ΔG^diss^ of ~6 kcal/mol) (Supplementary Fig. 10). Thus, REC-1 domains of hHK6 and Cal_Sln1 seem to show oligomerization states as dimers, or even trimers for hHK3, but their phosphorylation stabilizes the monomeric species suitable to interact with Ct_HPt. This feature contrasts with the phosphorylation-induced oligomerization observed in bacterial RRs^32^. Thus, we envision that reversibility could be prevented by subtle conformational changes in the REC-1 domain upon phosphoryl transfer, together with subtle conformational changes in the phosphorylated form of HPt. Both changes could contribute to lowering the affinity of the complex facilitating its release to ensure downstream signaling. In relation to HPt, we have observed subtle changes in the phosphomimetic mutant H105E. Meanwhile, the structure of Sc_Ypd1:REC_Ssk1_ (PDB:5KBX) shows a sulfate ion bound to the phosphorylatable His (stabilized by Q86 in Ypd1 and Q556 in Ssk1)^31^ that clashes with loop β4–α4 of REC_Sc_Sln1_ in the complex (PDB:2R25), and superposed REC_hHK6_, explaining why Ssk1 has to reorient to bind Sc_Ypd1. In relation to REC-1 domains, we have observed conformational changes located basically in loops β4–α4 and α4, thus, minimizing conformational changes in all loops during the recognition event for binding and release.

Overall, our studies propose that signaling from many hHKs to one HPt occurs by transient phosphorylation of the REC-1 domain which could be influenced by the absence of a Leu-Thr switch in β4, particularly effective for phosphotransfer when the binding affinity is low, which could contribute to a reduction, in recognition specificity. Contributing to reduced recognition specificity for binding REC-1 domains to HPt is the fact that interacting residues are rather conserved and are distributed in different areas promoting a compensatory effect to reduce selective pressure. At the same time, residues in HPt involved in interactions with REC-1 domains are also rather conserved between fungal species included in this study, which demonstrates reduced residue coevolution.

Finally, our structural studies on fungal HPt have revealed that they are decorated with N-terminal extensions and/or long loops αD–αE which could provide additional functions, such as phosphorylation stability and protein–protein interactions, as it has been observed in the case of *S. pombe* and *C. neoformans*^27,28^. In Cal_Ypd1, the long loop αD–αE is extended in contrast to Sc_Ypd1 which is constrained towards the helical bundle and this long loop does not seem to be involved in phosphotransfer as its deletion did not affect the velocity of the reaction. Thus, this loop could have additional functions such as being involved in interactions with other partners yet unknown. Alternative splicing has been observed in Ypd1 from *Magnaporthe oryzae* providing an explanation for isoforms with additional molecular mechanisms of signaling^57^. Finally, our studies have uncovered two salt bridges in Ct_HPt between two Glu residues in αB and an Arg residue in αE that provide bundle stability. As these residues seem rather conserved between human fungal pathogens, except in *S. cerevisiae*, we envision that these salt bridges could be present in other HPt proteins. These salt bridges do not intervene in complex recognition but seem to provide bundle stability preventing conformational plasticity.

## Methods

### Cloning and mutagenesis

Cloning of Ct_HPt (residues 9–175) and Cal_Ypd1 (residues 12–184) was done in vector LIC 1.4 (pETNKI-Strep3C-LIC-kanamycin) containing N-terminal Streptag^58^. Later, Ct_HPt (residues 9–175) was also cloned in vector LIC 1.1 (pETNKI-Strep3C-LIC-ampicillin) containing N-terminal 6× Histag for MST experiments. Meanwhile, REC-1 domains from hHK of *C. thermophilum* and hHK Sln1 of *C. albicans* were cloned in pLIC-SGC1 (TEV-ampicillin) containing N-terminal 6× Histag. Site-directed mutagenesis in Ct_HPt and REC-1 domains was done with the Q5® site-directed mutagenesis kit (New England Biolabs). Deletion mutant Cal_Ypd1 ∆107–147 was generated with InFusion® HD Cloning Kit (Takara Bio). A comprehensive list of constructs and primers used is shown in Supplementary Table 6.

### Expression and protein purification

For protein expression, *E. coli* Shuffle-T7 strain was used, which contains a chromosomal copy of disulfide bond isomerase DbsC to assist in the formation of correctly folded multi-disulfide bonded proteins. All strains containing the appropriate vector were grown on Hyper broth (Molecular Dimensions Ltd.) till the exponential phase (OD_600nm_ ∼ 0.6), then, induced with 0.5 mM isopropyl β-D-1-thiogalactopyranoside. Subsequently, REC-1 domains were incubated overnight at 20 °C while Cal_Ypd1 variants were incubated for 3 h at 37 °C. Finally, cells were centrifuged and stored at −20 °C. Expression conditions for each construct were determined as indicated in Supplementary Table 7.

For purification, cells expressing Streptag-Ct_HPt, or its mutants, were resuspended in buffer A (50 mM Tris pH 8.0, 150 mM NaCl) and sonicated after the addition of 1 mM of the protease inhibitor PMSF and 0.5 mM of the reducing agent TCEP. Then, cells were centrifuged (15,000×g, 4 °C) and the supernatant was loaded into a HiTrap CaptoQ column (Cytiva) to perform anion exchange chromatography, as the Streptag did not bind to the StrepTrap column. A gradient of 20 column volumes from buffer A to buffer B (50 mM Tris pH 8.0, 1 M NaCl) was performed and fractions enriched with Ct_HPt were dialyzed overnight at 4 °C with PreScission protease added in a molar ratio 1:1/20 (protein:protease) to remove the Streptag. Then, Ct_HPt was purified again by anion exchange chromatography and the protease was collected in the non-retained fraction, whereas the digested fraction was collected from the eluted fractions. At last, gel filtration chromatography was conducted in a 120 ml ProteoSEC 6/600 HR 16/60 (Generon) equilibrated in buffer C (50 mM Tris pH 8.0, 300 mM NaCl, 10 mM MgCl_2_). Protein fractions were concentrated to 10–30 mg/ml, frozen with N_2_(l), and stored at −80 °C.

Streptag-Cal_YPD1 WT and the mutant ∆107–147 were expressed as inclusion bodies, thus, after sonication and centrifugation, the pellet was resuspended in buffer A containing 2 M urea, then, incubated for 30 min at 4 °C shaking vigorously, then frozen at −20 °C overnight. The next day, cells were thawed, and centrifuged (15,000×g, 4°C) and the supernatant was subjected to anion exchange chromatography as explained for Ct_HPt.

For Histag-REC-1 domains, REC_hHK4_, REC_hHK5_, REC_hHK11_, REC_hHK6_, REC_hHK3_, and mutants, as well as REC_Cal_Sln1_, were resuspended in Buffer D (50 mM Tris pH 8.0, 500 mM NaCl, and 10 mM MgCl_2_), sonicated and centrifuged as stated before. Because REC-1 domains from *C. thermophilum* were mostly in inclusion bodies, pellets obtained after centrifugation were resolubilized as indicated in Cal_YPD1. Then, supernatants with soluble REC-1 domains were loaded into a HisTrap HP column (Cytiva) to perform affinity chromatography. Elution of the protein was achieved in buffer D containing 200 mM imidazole.

To remove the affinity tags of REC-1 domains and Cal_Ypd1 variants, the eluted proteins were dialyzed against buffer A and, at the same time, REC-1 domains were incubated with 10× Histag-TEV protease while Ca_Ypd1 variants were incubated with GST-PreScission protease, all in a molar ratio 1:1/20 (protein:protease). Then, the proteins were purified again by affinity chromatography with a HisTrap column for REC-1 domains and a HisTrap HP followed by a GSTrap column for Cal_Ypd1 variants. The digested protein was collected in the non-retained fraction separated from the bound non-digested fraction and the protease. Finally, the proteins were purified additionally with gel filtration chromatography using a ProSEC 16/60 6–600 HR column (Generon). Elution was performed in Buffer C, and proteins were concentrated, and stored. REC_hHK11_, due to its higher pI 9, was resuspended in Buffer E (50 mM Hepes pH 7.0, 500 mM NaCl, and 10 mM MgCl_2_) and purified by gel filtration chromatography in buffer F (50 mM Hepes pH 7.0, 300 mM NaCl, 10 mM MgCl_2_).

For Histag-Ct_HPt, the cell pellet was resuspended in Buffer D, sonicated, and centrifuged. The supernatant was loaded into a HisTrap HP column (Cytiva) to perform affinity chromatography and elution was achieved in buffer D containing 200 mM imidazole. Finally, the eluted fraction was dialyzed against buffer A and loaded into a HiTrap CaptoQ column to perform anion exchange chromatography, using a gradient of 20 column volumes from buffer A to buffer B (50 mM Tris pH 8.0, 1 M NaCl).

### Sequence alignment, protein modeling, and interaction surface determination in silico

For sequence alignment of proteins and residue conservation, we used the Praline Server^59^. For protein modeling, we used AlphaFold2^35^ and RobeTTAFold^37^ as deep-learning modeling methods. For interaction surface determination, with either solved or predicted structures, EPPIC Server^60^ and PISA Server^61^ were used. Figures for proteins were made using USCF Chimera^62^ and superpositions were performed using programs from CCP4 suite^63^.

### PAM synthesis

PAM was synthesized as described^34^. Briefly, phosphoryl chloride (4.6 ml) is added dropwise with vigorous stirring, for about 5 min, to 75 ml of an ice-cold 10% (*v*/*v*) aqueous ammonia solution. Fuming and heat are formed till a clear solution is obtained after about 15 min. The solution is diluted with 250 ml of acetone and two layers are formed. The bottom layer is separated and neutralized to approximately pH 6 (use pH test strip) with ~2 ml of glacial acetic acid. Then, the solution is left refrigerated overnight to induce crystallization. The next day, the solution is diluted with the same volume of ethanol at 96% (*v*/*v*) (75 ml) and further amounts of the salt are obtained. The product is filtered under vacuum and washed three times with ethanol at 96% (*v*/*v*), and then, air-dried.

### Oligomeric state determination by SEC-MALS

For the analysis of the oligomeric state, SEC-MALS experiments were performed using a Shimadzu HPLC with a UV detector (Shimadzu, 280 nm) coupled with a MALS detector (TREOS II, Wyatt Technology), a dRI detector (Optilab T-rEX, Wyatt Technology) and a DynaPro NanoStar® (Wyatt Technology). For the analysis of Cal_Ypd1 WT, Ct_HPt WT, and R158A, size exclusion chromatography was performed by injecting 40 µg of protein in a PROTEIN KW-803 (Shodex) column using a flow rate of 0.45 ml/min and a mobile phase consisting of 50 mM Hepes, pH 7, and 150 mM NaCl. For the analysis of the oligomeric state upon phosphorylation with PAM or BeF_3_^−^ conducted for REC_hHK6_ alone and with Ct_HPt, for REC_hHK3_ and REC_hHK5_, size exclusion chromatography was performed injecting 20 µg of protein in a PROTEIN KW-403 4 F (Shodex) column using a flow rate of 0.35 ml/min and a mobile phase consisting of 50 mM Hepes pH 7 and 150 mM NaCl. Data processing and MW calculations were carried out using ASTRA 7.1.2 software (Wyatt Technology).

### Structural stability assays

The stability of Ct_HPt variants proteins was assessed using the temperature-induced protein unfolding assay called Thermofluor. The fluorescent dye SYPRO Orange® (Sigma Aldrich) at 10× final concentration was mixed with protein at a final concentration of 25 μM in Buffer C. The assay was performed in a CFX96 real-time PCR Detection System® with a C1000 Thermal Cycler (BioRad) from 20 °C to 85 °C for the detection of the FRET channel (excitation wavelength between 450 nm and 490 nm and emission wavelengths between 560 nm and 580 nm). Data were processed by plotting the negative derivative of the fluorescence signal vs time against the temperature to determine the Tm.

### Native gel phosphorylation and phosphotransfer experiments

For phosphotransfer, 3 μg (~20 µM) of REC-1 domain was incubated for 30 min at 37 °C with 50 mM PAM or AcP in 1× phosphorylation buffer (50 mM Tris pH 8.0, 10 mM KCl, 150 mM NaCl, 10 mM MgCl_2_). Then, 3 μg (~16 µM) of Ct_HPt/Cal_Ypd1 was added in a total reaction volume of 10 μl and samples were taken at different time points and different temperatures, 37 °C, (RT) and 4 °C, depending on the experiment. In the case of Ct_HPt, screening against REC-1 domains was done incubating at 30 min final time. Phosphotransfer at different time points with each REC-1 domain incubated with Ct_HPt WT or H105E was performed during 0.5 min, 1 min, and 5 min. Meanwhile, for phosphotransfer experiments from REC_Cal_Sln1_ to Cal_Ypd1, upon incubation, samples were taken at 1 min, 5 min, and 15 min. Phosphotransfer from hHK6_691-end to Ct_HPt was performed by incubating 5 μg (8 μM) of the hHK hHK6_691-end for 30 min at 37 °C with 5 mM ATP in 1× phosphorylation buffer. Then, 3 μg (~16 µM) of Ct_HPt WT or H105E was added, in a total reaction volume of 10 µl, and samples were taken at 1 min, 5 min, 10 min, and 15 min upon incubation. Finally, phosphotransfer experiments performed with decreasing concentrations of REC-1 were conducted by mixing with HPt for 0.5 min for all samples. Controls of Ct_HPt/Cal_Ypd1 phosphorylation were assessed by incubating 3 μg (20 µM) of protein with 50 mM PAM for 30 min. In the case of Cal_Ypd1 variants, phosphorylation was performed by adding 10 mM DTT to the phosphorylation buffer.

After phosphotransfer and phosphorylation experiments, 3 μl of 5× native loading buffer (0.5 M Tris pH 6.8, 87% glycerol, 0.5% bromophenol blue) containing 50 mM EDTA was added at each time point in the 10 μl volume sample and was loaded into a 12% (for Ct_HPt) or 15% (for Cal_YPD1) native PAGE gel. Then, the gel was run in a native running buffer (25 mM Tris pH 8.0, 192 mM Glycine) at 150 V for 1 h and 30 min at 4 °C. Native PAGE gels were stained with Coomassie blue solution and destained with distilled water for their visualization. Due to the high pI (8.51) of hHK hHK6_691-end, phosphotransfer experiments were run in blue native gels, which used a 5× blue native loading buffer (SERVA), a cathode running buffer (50 mM Tricine, 10 mM BisTris) and an anode running buffer (50 mM BisTris pH 7.0). The blue native gels were run for 10 min at 50 V first and 2 h at 200 V, then, gels were stained and destained as the aforementioned method.

Quantification of phosphorylation was carried out with GelAnalyzer 23.1.1 (available at www.gelanalyzer.com).

### Radioactivity assays

To assess phosphotransfer from REC-1 to Ct_HPt, phosphorylation of REC-1 domains with [^32^P]-AcP was performed in the first place. For that purpose, [^32^P]-AcP was synthesized by incubating for 2 h at RT 2.5 U of acetate kinase with 10 µl of 1 μCi/μl of [γ-^32^P] ATP (1000 Ci/mmol Perkin Elmer) in 2.5 mM Tris pH 8, 6 mM potassium acetate and 1 mM MgCl_2_ buffer in a final volume of 100 µl. Then, the mixture was centrifuged (14,000× g, 30 min) with a Microcon-10 kDa Centrifugal Filter Unit (GE Healthcare) to eliminate the acetate kinase. Phosphorylation of REC-1 was performed by incubating 1 mg/ml (~50 µM) final protein concentration with 8.8 µl of [^32^P]-AcP synthesized in a solution containing 50 mM Tris-HCl pH 8, 100 mM KCl, 10 mM MgCl_2_, and 150 mM NaCl, with a total volume of 50 μl. Then, 20 μl were transferred to a new tube containing 1.4 μl of Ct_HPt 14 mg/ml (50 µM) and the reaction was stopped after 15 and 45 min, adding 8 μl of loading buffer containing 50 mM EDTA and 4% SDS to 8 μl of sample. Control samples of REC-1 domain without Ct_HPt were also collected after 15 min and 45 min and stopped as indicated. The samples were loaded in a 15% SDS-PAGE gel and run at 150 V at RT. Phosphorylated proteins were visualized by phosphorimaging using a Fluoro Image Analyzer FLA-5000 (Fuji) and processed with the MultiGauge software (Fuji).

### MST

The interaction of REC-1 domains with Ct_HPt was assessed by MST. Two hundred microlitres of 0.2 µM Histag-Ct_HPt was mixed with 200 µl of Red-Tris NTA Dye and incubated for 30 min at RT, and then centrifuged for 10 min at 15,000 g. Then, each REC-1 domain was diluted by performing sixteen serial dilutions by a factor of two decreasing concentrations from 1 mM REC_hHK3_, 1 mM REC_hHK5_, 1 mM REC_hHK3_ N1107A/N1110A, and 0.32 mM REC_hHK6_ in PBS-T (PBS + 0.05% (*v*/*v*) Tween-20). Subsequently, 10 µl of each dilution was mixed with 10 µl of labeled Ct_HPt and loaded into glass capillaries Standard Monolith Tubes (K005, NanoTemper Technologies). Samples were excited with the Nano-RED detector at medium MST power and automatic excitation. Data were collected in the Monolith 2020 TNG instrument (MM026, NanoTemper Technologies). The fluorescence profile is registered for several seconds before turning on the infrared laser, for 21 s from the moment the infrared laser turns on, and finally for 4 s after the laser turns off (to corroborate the return of the fluorescence toward the initial values). A previous run was done with the labeled Ct_HPt alone to determine the efficiency of the labeling.

Binding curves correspond to hyperbolic fitting (in semilog representation) of the fractional fluorescence change arising from fluorescently labeled Ct_HPt at different concentrations of diluted REC-1 domains. The fraction of saturation estimated for each concentration of diluted REC-1 domain corresponds to the quotient (Fx − F0)/(F∞ − F0), where F0, Fx, and F∞ are the fluorescence in the absence, at a given concentration, and at infinite concentration of the REC-1 domain that is varied, respectively. F∞ was estimated from the hyperbolic fitting. The *K*_*D*_ values are the concentrations giving a half-maximum change. Each point is the mean for two different titrations. Adjustments to curves and *K*_*D*_ were calculated with the M.O. Affinity analysis software (Nanotemper Technologies) and represented with GraphPad Prism 8.

### Protein crystallography

Crystals were obtained using the sitting drop vapor diffusion technique. Crystallization of Ct_HPt was achieved by mixing 0.3 μL of a solution containing 10 mg/ml of protein with 0.3 µl of different screening solutions (JBScreen Classic HTS I and HTS II, Jena Bioscience). Crystals were grown in 1.5 M sodium citrate pH 6.5, however, better diffracting crystals were obtained from a random microseed matrix screening^64^. The same screening assay was performed, and crystals grew in several conditions, including 1.5 M sodium citrate pH 6.5 where crystals were collected and diffracted X-ray to 2.4 Å resolution. For cryopreservation, the crystals were briefly passed through a solution with 1.5 M sodium citrate pH 6.5 and 12% (*v*/*v*) ethylene glycol. Crystallization of the complex REC_hHK6_:Ct_HPt was achieved by mixing 0.3 µl of a solution containing 10 mg/ml of REC_hHK6_ with 10 mg/ml HPt, 7 mM MgCl_2_, 5 mM BeSO_4_, and 30 mM NaF with 0.3 µl of screening solutions. Crystals grew in a condition with 1.6 M ammonium sulfate and 1 M lithium sulfate, and were collected passing them briefly through a cryoprotectant solution containing 2.6 M lithium sulfate. Then, crystals diffracted X-ray up to 2.4 Å resolution. Crystallization of REC_hHK3_ was achieved by mixing 0.3 µl of a solution containing 10 mg/ml of protein, 7 mM MgCl_2_, 5 mM BeSO_4_, and 30 mM NaF with 0.3 µl of the screening solutions. Crystals were obtained in a condition with 2 M ammonium sulfate and 0.1 M Tris pH 8.5, then, they were cryopreserved in 2 M lithium sulfate and diffracted X-ray to 1.9 Å resolution. Crystallization of REC_Cal_Sln1_ was achieved by preparing the same sample as for REC_hHK3_ and crystals were grown in a condition with 30% (*v*/*v*) PEG 4000, 0.1 M Tris pH 8.5, and 0.2 M of MgCl_2_. They were harvested directly without cryoprotectant and diffracted up to 1.5 Å resolution. Crystallization of Ct_HPt H105E was achieved by preparing the same sample as the wild-type and crystals were grown in a condition containing 0.5 M ammonium dihydrogen phosphate and 0,2 M sodium citrate. Crystals were harvested by passing them briefly through a solution of 35% ethylene glycol and diffracted X-ray to up to a 2.4 Å resolution. Finally, crystallization of Ct_HPt R158A was achieved by preparing the same sample as the wild-type, and crystals grew in a condition containing 1.6 M ammonium sulfate and 1 M lithium sulfate. Crystals were collected by passing them briefly through a solution of 2 M lithium sulfate and diffracted X-ray up to a 3.4 Å resolution. Diffraction and data collection for the crystals was conducted in the I03 beamline of Diamond Light Source Synchrotron (Didcot, UK) for Ct_HPt and in the BL13-XALOC of Alba Synchrotron (Cerdanyola del Vallès, Spain) for the complex and REC-1 domains. Datasets with the highest resolution were used to solve the structures. Data integration and reduction were performed with XDS^65^ and Aimless from the CCP4 suite^63^. Molecular replacement was conducted with Balbes^66^ for Ct_HPt, REC_hHK3_ and REC_Cal_Sln1_ and Phaser^67^ was used for REC_hHK6_-BeF:Ct_HPt using the solved structure of Ct_HPt and a Robetta-modeled REC_hHK6,_ and also for Ct_HPt H105E and Ct_HPt R158A using the solved structure of Ct_HPt. The definitive structural models were obtained by iterative cycles of tracing with Coot^68^ and refining with Refmac5^69^. Data collection and refinement statistics are included in Table 1 and Supplementary Table 4. The Ramachandran plot for refined Ct_HPt showed 99.06% residues in favored region, 1.75% in allowed region and 0.19% of outliers; for refined REC_hHK3_ it showed 94.26% residues in favored region, 2.02% in allowed region and 0.40% of outliers; for refined REC_hHK6_-BeF:Ct_HPt complex showed 97.58% residues in favored region and 4.12% in allowed region; for REC_Cal_Sln1_, it showed 97.18% residues in allowed region, 2.21% in allowed regions and 0.60% of outliers; for refined Ct_HPt H105E it showed 98.44% residues in favored regions, 1.56% in allowed regions, and 0% of outliers; and finally for refined Ct_HPt R158A it showed 91.02% residues in favored region, 8.59 in allowed region and 0.39% of outliers.

### SEC-SAXS experiment

SAXS data were collected at ESRF, Grenoble (France), using SAXS beamline BM29 with a wavelength of 0.99 Å on a Pilatus 2 M detector (DECTRIS) at 20 °C. For SEC-SAXS, 50 µl of Cal_Ypd1 at 8 mg/ml were injected onto a Superdex 75 Increase 3.2/300 column (equilibrated in 50 mM Tris-HCl pH 8, 300 mM NaCl) at a flow rate of 70 µl/min. Scattering data were acquired as components eluted from the column and passed through the SAXS measuring cell. The ATSAS software package^40^ was used to normalize the data to the intensity of the incident beam, to average the frames, and to subtract the scattering contribution from the buffer. The Rg, maximum particle dimension (*D*_max_), and distance distribution function (*p*(*r*)) were evaluated using the program PRIMUS as part of the ATSAS package. A model was generated using the program DAMMIF in the ATSAS package and the final model was identified by DAMAVER. Finally, a model structure for Cal_Ypd1, obtained via the Robetta server^37^ was modeled against the processed SAXS data with rigid body refinement using SASREF included in the ATSAS online server.

### Statistics and reproducibility

For phosphotransfer experiments using native gels and calculation of affinity values for complex formation using thermophoresis, at least two experiments were performed.

### Supplementary information

Supplementary Information

Description of Additional Supplementary Files

Supplementary Data 1

## Data Availability

The X-ray crystallographic coordinates reported for all the structures have been deposited at the Protein Data Bank. For Ct_HPt, the PDB accession code is 8PBW, for complex REC_hHK6_-BeF:Ct_HPt is 8PDC, for REC_hHK3_ is 8PHN, for REC_Cal_Sln1_ is 8HPX, for Ct_HPt H105E is 8RQG, and for Ct_HPT R158A is 8RQJ. The SAXS data for Cal_Ypd1 has been deposited in the Small Angle Scattering Biological Data Bank under the code SASDSU5. Supplementary Figs. 1–17 and Tables 1–7 are provided as a Source data file. The numerical source data for Figs. 2b and 6d can be accessed through Supplementary Data 1 Excel file. The authors declare that all other relevant data supporting the findings of this study are included in this published article and its Supplementary information files, or from the corresponding authors upon request.
