# Supplementary material for: Structural and functional insights underlying recognition of histidine phosphotransfer protein in fungal phosphorelay systems
Source: Commun Biol. 2024 Jul 4;7:814. doi: 10.1038/s42003-024-06459-0 (PMC11224324; doi:10.1038/s42003-024-06459-0)
Supplement: Supplementary file 1 — Supplementary Information [file 42003_2024_6459_MOESM1_ESM.pdf]

Structural and functional insights underlying recognition of Histidine phosphotransfer protein in fungal phosphorelay systems

Francisco Paredes-Martínez, Lluís Eixerés, Sara Zamora, Patricia Casino

Table of contents

Supplementary Tables 1-7

Supplementary Fig. 1-17

Supplementary Table 1. Interacting residues in the REC_{hHK6}-BeF:Ct_HPt complex.

**Ct_HPt**	**REC_{hHK6}**	**Distances (Å)**
Polar interactions		
80(GLN) in αB<br>106(PHE) in αC<br>110(SER) in αC	1173(ASN) in Loop β1-α1	2.96<br>2.92<br>3.36
76(ASN) in αB	1174(THR) in α1	3.09
109(GLY) in αC<br>113(THR) in αC	1176(ASN) in α1	3.18<br>2.81
69(PHE) in αB	1182(ARG) in α1	3.33
108(LYS) in αC	1250(ALA) in Loop β4-α4	2.64
127(GLN) in αD<br>108(LYS) in αC	1252(SER) in Loop β4-α4	2.50<br>2.94
57(GLN) in αA<br>54(ILE) in αA<br>113(THR) in αC	1274(ARG) in Loop β5-α5	2.65<br>3.02<br>2.79
Hydrophobic interactions		
106(PHE) in αC	1172(ASP) in Loop β1-α1<br>1173(ASN) in Loop β1-α1	3.77<br>3.54
80(GLN) in αB<br>110(SER) in αC	1173(ASN) in Loop β1-α1	3.92<br>3.89
110(SER) in αC<br>73(LEU) in αB<br>80(GLN) in αB<br>77(PHE) in αB	1175(VAL) in α1	3.68<br>3.70<br>3.77<br>3.95
113(THR) in αC<br>73(LEU) in αB	1179(VAL) in α1	3.70<br>3.54
69(PHE) in αB	1182(ARG) in α1	3.66
105(HIS) in αC	1223(GLN) in Loop β3-α3<br>1250(ALA) in Loop β4-α4	3.68<br>3.67
113(THR) in αC	1272(PRO) in Loop β5-α5	3.79
61(MET) in αA<br>57(GLN) in αA	1274(ARG) in Loop β5-α5	3.90<br>3.58

Supplementary Table 2. Conservation in nature of interacting residues at REC_{hHK6} in other REC domains.

**REC_{hHK6}**	**REC_{hHK3}**	**REC_{hHK5}**	**REC_{hHK4}**	**REC_{hHK11}**	**REC_{Cal_Sln1}**	**REC_{Sc_Sln1}**
1172(ASP)	Asp	Asp	Asp	-	Asp	Asp
1173(ASN)	Asn	Asn	Asn	-	Asn	Asn
1174(THR)	Ala	Pro	Leu	-	Ser	His
1175(VAL)	Val	Val	Ile	-	Val	Val
1176(ASN)	Asn	Asn	Asn	-	Asn	Asn
1179(VAL)	Leu	Val	Ile	-	Val	Val
1182(ARG)	Lys	Lys	Arg	Lys	Arg	Arg
1223(GLN)	Gln	His	Glu	Glu	Gln	Gln
1250(ALA)	Ala	Ala	Ala	Ala	Ala	Ala
1252(SER)	Ala	Val	Ala	Ala	Ala	Ala
1272(PRO)	Pro	Pro	Pro	Pro	Pro	Pro
1274(ARG)	Gln	Asp	Arg	Gln	Ser	Lys

*Non conserved residues are highlighted in yellow

Supplementary Table 3. Conservation in nature of interacting residues at Ct_HPt in other fungal HPt domains.

**Ct_HPt**	**Cal_Ypd1**	**Cau_Ypd1**	**Af_Ypd1**	**Hc_Ypd1**	**Bd_Ypd1**	**Cn_Ypd1**	**Nc_HPt**	**Sc_Ypd1**
54(ILE)	Val	Val	Thr	Thr	Thr	Thr	Ile	Ile
57(GLN)	Glu	Glu	Gln	Gln	Gln	Gln	Gln	Glu
61(MET)	Met	Met	Met	Met	Met	Met	Met	Met
69(PHE)	Phe	Phe	Phe	Phe	Phe	Phe	Phe	Phe
73(LEU)	Leu	Leu	Ile	Leu	Leu	Ile	Leu	Leu
76(ASN)	Val	Thr	Gly	Gly	Gly	Gly	Asn	Gln
77(PHE)	Phe	Phe	Phe	Phe	Phe	Phe	Phe	Phe
80(GLN)	Gln	Gln	Gln	Gln	Gln	Gln	Gln	Gln
105(HIS)	His	His	His	His	His	His	His	His
106(PHE)	Phe	Tyr	Phe	Phe	Phe	Phe	Phe	Phe
108(LYS)	Lys	Lys	Lys	Lys	Lys	Lys	Lys	Lys
109(GLY)	Gly	Gly	Gly	Gly	Gly	Gly	Gly	Gly
110(SER)	Ser	Ser	Ser	Ser	Ser	Ser	Ser	Ser
113(THR)	Ala	Ala	Thr	Thr	Thr	Ala	Thr	Ala
127(GLN)	Gln	Gln	Gln	Gln	Gln	Gln	Gln	Gln

*Non conserved residues are highlighted in yellow. Acronyms are: Cau_Ypd1 for *C. auris*; Af_Ypd1 for *A. fumigatus*; Hc_Ypd1 for *H. capsulatum*; Bd_Ypd1 for *B. dematitidis*; Cn_Ypd1 for *C. neoformans*; Nc_Ypd1 for *N. crassa*

Supplementary Table 4. Data collection and refinement statistics for the obtained structures of Ct_HPt H105E and Ct_HPt R158A.

	**Ct_HPt H105E**	**Ct_HPt R158A**
**Data collection**		
Space group	$P6_4$ 2 2	F 2 2 2
Cell dimensions		
a, b, c (Å)	100.58 100.58 65.27	122.35 123.16 128.91
α, β, γ (º)	90.00 90.00 120.00	90.00 90.00 90.00
Resolution (Å)	87.10-2.40 (2-49-2.40)	72.00-3.40 (3.67-3.40)
No. reflections	412619 (33013)	87635 18459
R_{sym} or R_{merge}	0.201 (2.112)	0.258 (1.971)
R_{pim}	0.028 (0.335)	0.076 (0.559)
I/σI	27.5 (2.7)	8.8 (2.4)
Completeness (%)	100 (100)	100 (99.9)
Redundancy	51.2 (40.0)	12.7 (13.3)
**Refinement**		
R_{work}/R_{free}	0.21/0.26	0.24/0.30
No. atoms		
Protein	1004	1824
Ligand/Ion	12	5
Water	16	8
B-factors		
Protein	54.57	121.79
Ligand/Ion	46.93	137.19
Water	53.65	86.55
R.m.s deviations		
Bond lengths (Å)	0.005	0.002
Bond Angles (º)	1.14	0.09
PDB Code	8RQG	8RQJ

Values in parentheses are for the highest-resolution shell.

Supplementary Table 5. Analysis of the active site configuration in several REC domains.

REC domains from RRs	PDB	Distance (Å) (O_{Leu}β4 with N_{Asp+1}β3)	Leu-Thr switch	Leu rotamer χ1 angle (°)	Y-T mechanism	Distance (Å) (F-N) (BeF_3- with Thr+1β3)
RR468	3DGF	3.1	No	-54	Inactive	
RR468 + BeF	3GL9	4.4	Yes	-174	Active	2.8
PhoB	1B00	3.1	No	-56	Inactive	
PhoB+BeF	1ZES	4.4	Yes	-173	Active	2.8
ArcA	1XHE	3.1	Intermediate	-115	Inactive	
ArcA+BeF	1XHF	4.6	Yes	-175	Active	2.9
KdpE	1ZH2	3.1	Ser-Leu, No	-60	Inactive	
KdpE+BeF	1ZH4	4.5	Ser-Leu, Yes	-150	Active	2.8
DrrB	1P2F	3.0	No	-57	Inactive	
DrrB+BeF	3NNS	4.0	Yes	-178	Active	3.1
CheY	1EHC	2.7	Val-Thr	-	Inactive	
CheY+BeF	1FQW	4.5	Val-Thr	-	Active	2.9
DesR-REC	4LE1	3.2	No	-55	Inactive	
DesR-REC+BeF	4LE0	4.3	Yes	-178	Active	3.0
REC_{Sc_Sln1}-Ypd1	1OXB	3.1	No	-43	Inactive	
REC_{Sc_Sln1}+BeF-Ypd1	2R25	4.7	Yes	-154	Active	2.7
REC_{hHK6}+BeF-HPt	8PDC	3.6	No	-53	Inactive	-
REC_{hHK3}	8PHN	3.1	No	-47	Inactive	
REC_{Cal_Sln1}	8PHX	3.1	No	-51	Inactive	
HK853-RR468+SO4 Phosphatase state	3DGE	4.2	Intermediate	-82	Active	3-3.4
DesK+DesR-REC+BeF Phosphatase state	7SSJ	3.2-3.3	Intermediate	-74/-54/-67	Active	3.2-3.5

Supplementary Table 6. Primers and vectors used.

Construct	Vector	Primers (5'-3')
**Ct_HPt** (9-175)	LIC 1.4	Fw: cagggacccggtGCTGATCGGGCGCAGGATC<br>Rv: cgaggagaagcccggttaATACTCAAAAAACTCACGCATCAT
E82A	LIC 1.4	Fw: TGAACAAGCAGCGGAAACCTTCC<br>Rv: AAAAAGTTGAGCACCAGTG
E89A	LIC 1.4	Fw: CCAGAAAATGGCGACGGCGCTGA<br>Rv: AAGGTTTCCTCTGCTTGTTC
R158A	LIC 1.4	Fw: GGCGAGCGCAGCGGTGGATACGG<br>Rv: AGCGCTTCCGCAATTTTT
R169A	LIC 1.4	Fw:TAAAATGATGGCGGAATTCTTTGAATACTAAAGCAAGCTGGGCCAC<br>Rv: TGCAGCGCCACCGTATCC
H105E	LIC 1.4	Fw: CAAGCTGGGCgaaTTTTTAAAAGG<br>Rv: CTCAACTCTGGCAGGTCT
**Cal_Ypd1** (12-184)	LIC 1.4	Fw: cagggacccggtTCAGGACTTGTCGACTGGGC<br>Rv: cgaggagaagcccggttaTTCGTAATATTCGTCCAATGCTC
Δ107-147	LIC 1.1	Fw: TTTCAATTGGAAGATATACCGGATGAATCAAGCGATGAC<br>Rv: ATCTTCCAATTGAAAATTGTCAAAGTTG
**REC_{hHK4}** (1289-1490)	pLIC-SGC1	Fw: tacttccaatccatgGAAGTATCAACTCCTCAAATTAGG<br>Rv: tatccacctttactgtcaCCGCATCTTCTCCAGCAACTCC
**REC_{hHK11}** (1421-1547)	pLIC-SGC1	Fw: tacttccaatccatgCCCACTCCCCCACTTACCCT<br>Rv: tatccacctttactgtcaTGCGGGGCCCTCGAGCA
**REC_{hHK5}**	pLIC-SGC1	Fw: tacttccaatccatgGGGAAGGGCTATCGGGTCTTACT

(1621-1892)		Rv: tatccacctttactgtcaGAAGAACTTCGCCAACGCCC
**REC$_{hHK6}$** (1152-1290)	pLIC-SGC1	Fw: tacttccaatccatgCAGGCCCTCACCATCAAACCG<br>Rv: tatccacctttactgtcaCTCCAATATCGTGGAGAACTTCGC
N1173A	pLIC-SGC1	Fw: CGCCGATGACGCGACTGTCAACATCGAG<br>Rv: ACAAGCACGCGGAGCTTC
N1173A N1176A	pLIC-SGC1	Fw: GTCGCGATCGAGGTCGTCTCGCGC<br>Rv: AGTCGCGTCATCGGCGACAAGCAC
T1249A	pLIC-SGC1	Fw: CGTCGCGCTGgcgGCCTTCTCGG<br>Rv: ATCGGCGCGTTGTAACCCAAGGC
**REC$_{hHK3}$** (1085-1220)	pLIC-SGC1	Fw: tacttccaatccatgGAAAACCGGGCAACGCCGTC<br>Rv: tatccacctttactgtcaCGCGCACTTGAGGATAGTCTGAA
N1107A	pLIC-SGC1	Fw: GGCCGAAGACGCGGCAGTCAACC<br>Rv: AGCAGAATCTCAAATGAC
N1107A N1110A	pLIC-SGC1	Fw: GTCGCGCAGCGGTTGGCTGTGAAG<br>Rv: TGCCGCGTCTTCGGCCAGCAGAATC
T1183A	pLIC-SGC1	Fw: TATCGCCCTCgcgGCGCACGCCA<br>Rv: ATTGGCGTGCGTTGTGGGG
**REC$_{Cal_Sln1}$** (1240-1368)	pLIC-SGC1	Fw: tacttccaatccatgGATATATCACATTTGAGAGTTTTAGTA<br>Rv: tatccacctttactgtcaTGATAAAAATTCAACAAGAACTTTC
T1329A	pLIC-SGC1	Fw: TATTGCCTTAgcgGCATTTGCTG<br>Rv: ATTGGTTTATTATATTGTAAATTTTTTC

Supplementary Table 7. Conditions of induction and solubility of REC-1 domains and HPt proteins.

Construct	Conditions of induction	Solubility
Ct_HPt	20ºC overnight	Soluble
Ct_HPt E82A	20ºC overnight	Soluble
Ct_HPt E89A	20ºC overnight	Soluble
Ct_HPt R158A	20ºC overnight	Soluble
Ct_HPt R169A	20ºC overnight	Soluble
Ct_HPt E82A R158A	20ºC overnight	Soluble
Ct_HPt H105E	20ºC overnight	Soluble
Cal_Ypd1	37ºC 3 h	Inclusion bodies
Cal_Ypd1 Δ107-147	37ºC 3 h	Inclusion bodies
REC$_{hHK4}$	20ºC overnight	Inclusion bodies
REC$_{hHK11}$	20ºC overnight	Inclusion bodies
REC$_{hHK5}$	20ºC overnight	Inclusion bodies
REC$_{hHK6}$	20ºC overnight	Inclusion bodies
REC$_{hHK6}$ N1173A	20ºC overnight	Inclusion bodies
REC$_{hHK6}$ N1173A N1176A	20ºC overnight	Inclusion bodies
REC$_{hHK6}$ T1249A	20ºC overnight	Inclusion bodies
REC$_{hHK3}$	20ºC overnight	Inclusion bodies
REC$_{hHK3}$ N1107A	20ºC overnight	Inclusion bodies
REC$_{hHK3}$ N1107A N1110A	20ºC overnight	Inclusion bodies
REC$_{hHK3}$ T1183A	20ºC overnight	Inclusion bodies
REC$_{Cal_Sln1}$	20ºC overnight	Soluble
REC$_{Cal_Sln1}$ T329A	20ºC overnight	Soluble

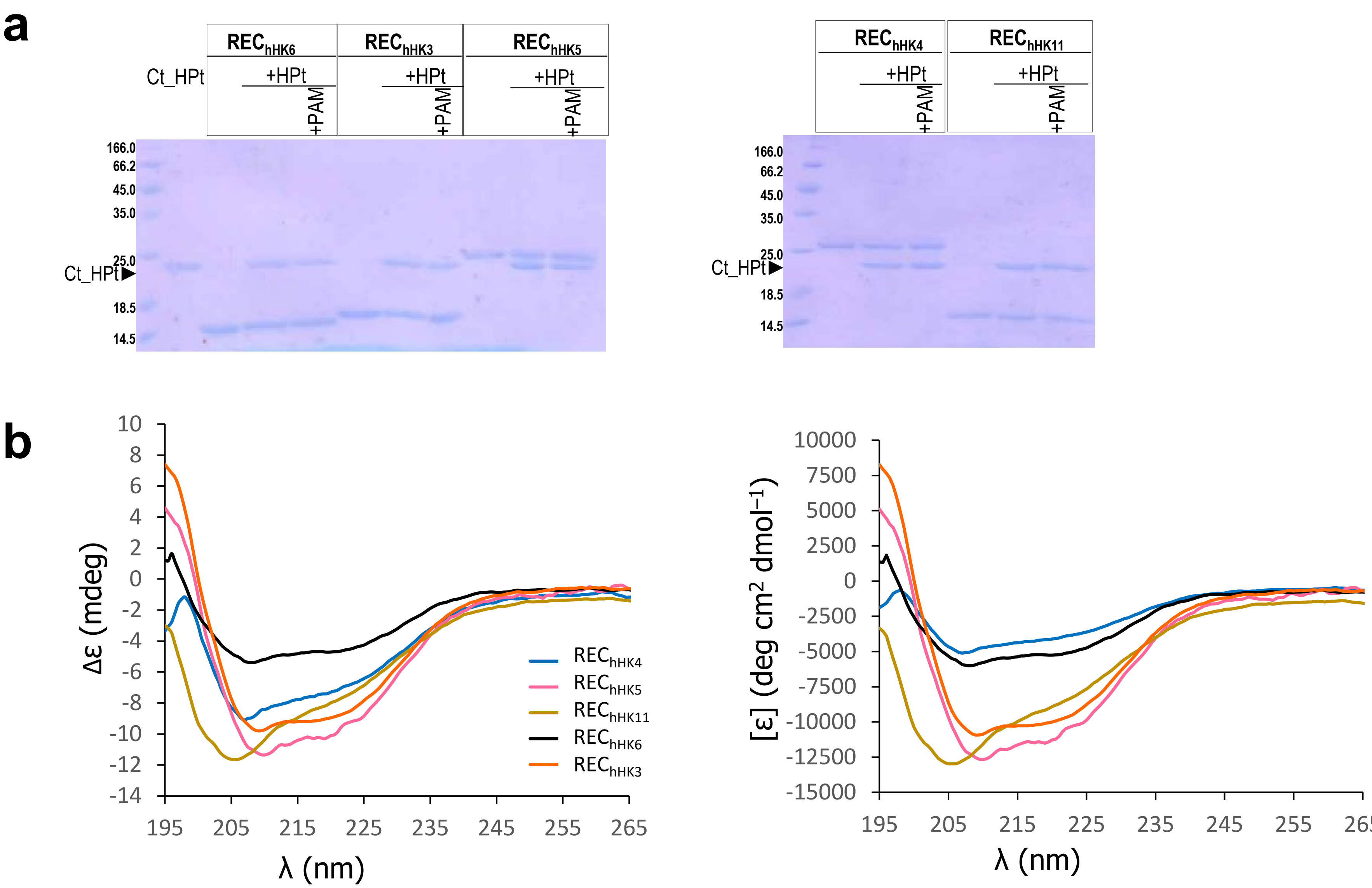

Supplementary Fig. 1. Purity and stability of REC-1 domains used in the phosphotransfer assays. a) SDS-PAGE gel for the samples used in the phosphotransfer experiments run in Native gels. b) Circular dichroism spectra for REC_{hHK4}, $REC_{hHK5,}$ REC_{hHK11}, REC_{hHK6} and REC_{hHK3}. represented as Δε (mdeg) versus wavelength (λ) in nm or as [ε] in deg cm^2 $dmol^{-1}$ versus λ (calculated as [ε] = (millidegrees x mean residue weight)/(pathlength in millimeters x concentration in mg/ml) according to Greenfield NJ. Nat Protoc. 2006;1(6):2876-90).

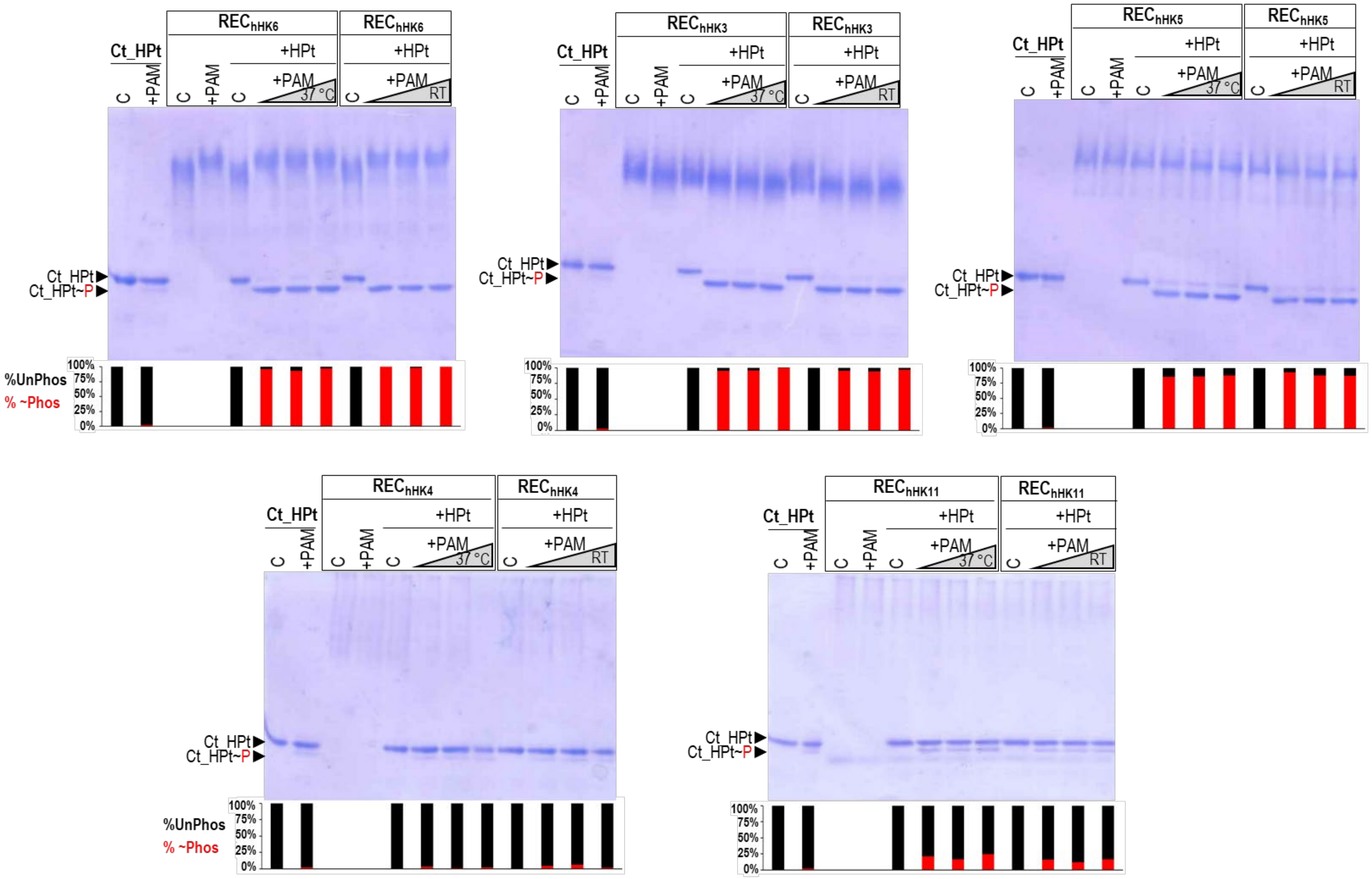

Supplementary Fig. 2. Phosphotransfer experiments from REC-1 domains phosphorylated with PAM to Ct_HPt. The REC-1 domains were phosphorylated during 30 min at 37 °C, then, Ct_HPt was added, and the mixture was incubated for 0.5, 1 and 5 min, either at room temperature (RT) and at 37 °C. For RT, the REC-1 domains phosphorylated were incubated at RT for 3 min to lower down the temperature prior to mixing it with Ct_HPt. The lines containing "C" refers to the proteins without phosphodonor.

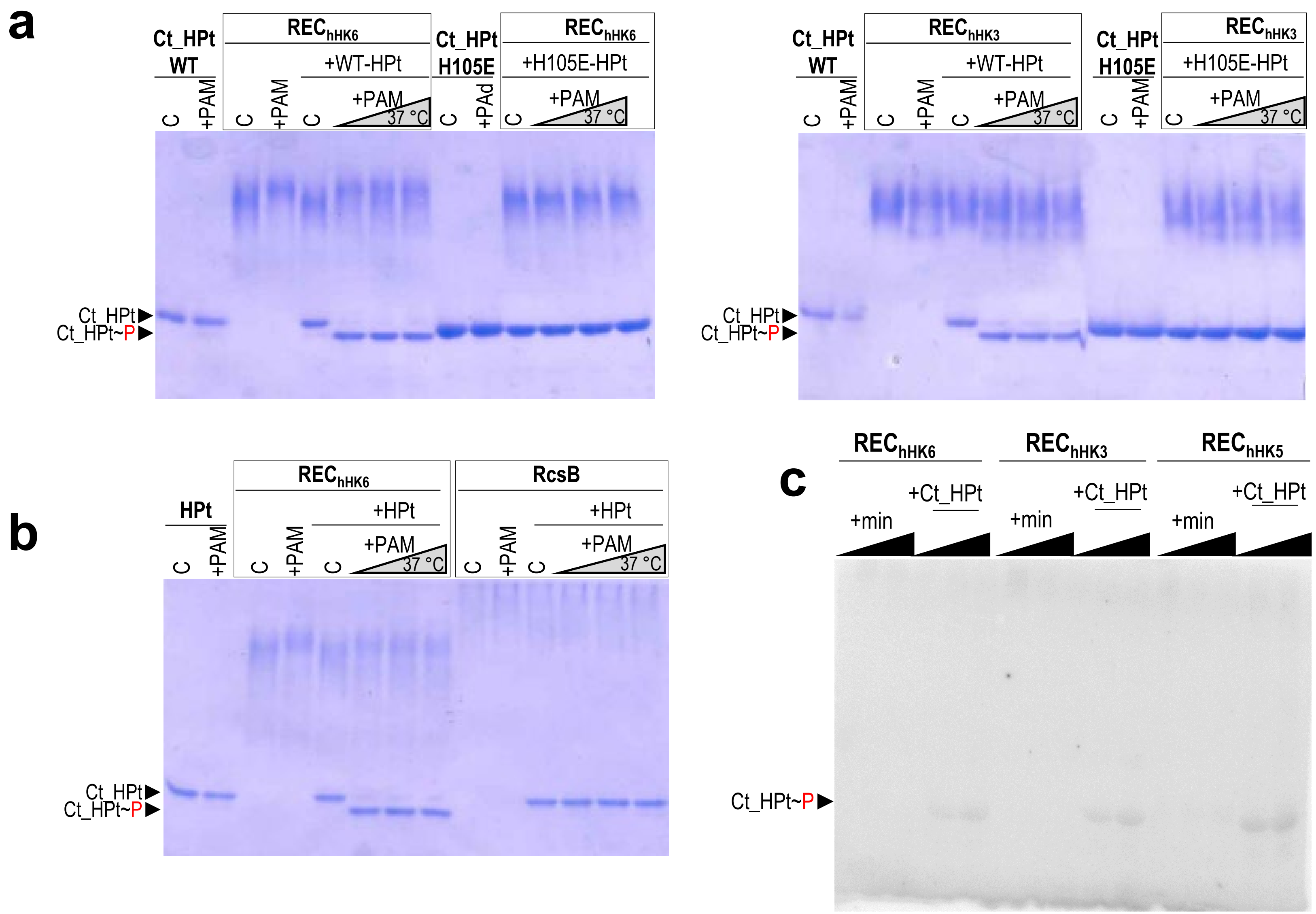

Supplementary Fig. 3. Native-gel phoshotransfer from REC-1 domains to Ct_HPt. a) Phosphotransfer experiments from REC_{hHK6}, or REC_{hHK3} to Ct_HPt WT or mutant H105E. REC-1 domains were phosphorylated with PAM during 30 min at 37 °C, and then phosphotransfer to Ct_HPt WT or mutant H105E was performed during 0.5, 1 and 5 min at 37 °C**.** b) REC_{hHK6} domain and RcsB were phosphorylated during 30 min at 37 °C, then, Ct_HPt was added and the mixture was incubated during 0.5, 1 and 5 min at 37 °C**.** c) Phosphotransfer experiments to Ct_HPt using REC-1 domains phosphorylated with [^{32}P]-AcP. First, REC_{hHK6}, REC_{hHK3} and REC_{hHK5} were incubated with 0,4 µCi/µl of [^{32}P]-AcP, and after 45 min, Ct_Hpt was added and phosphotransfer was stopped at 15 and 45 min. Control for REC-1 domain phosphorylation was collected after 15 and 45 min of reaction. The lines containing “C” refers to the proteins without phosphodonor.

a

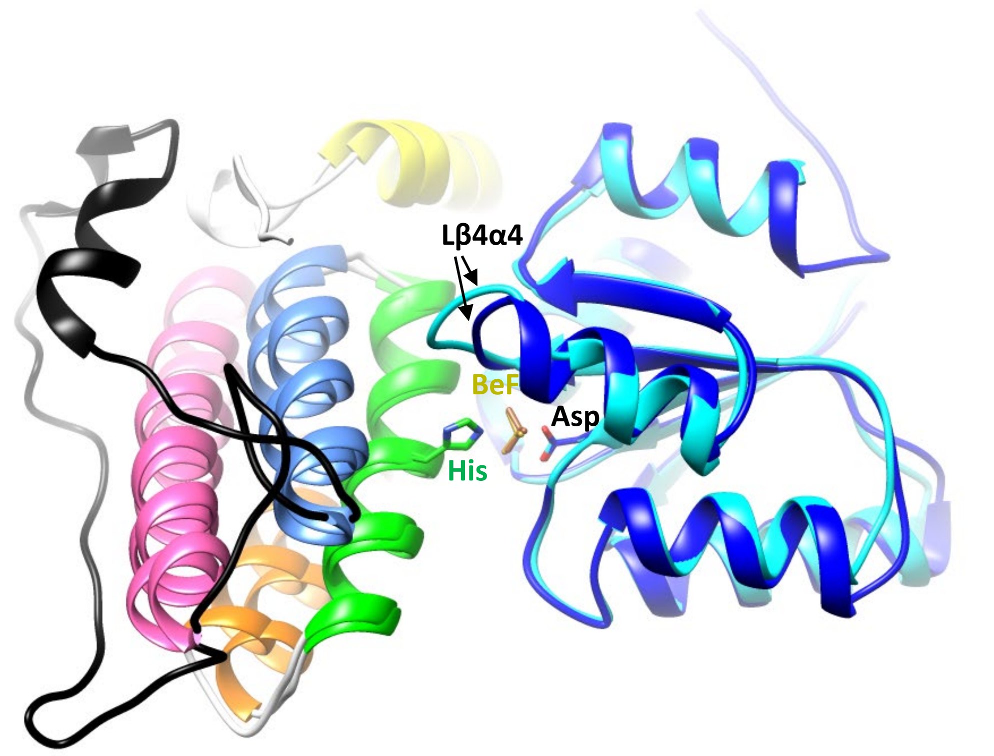

b

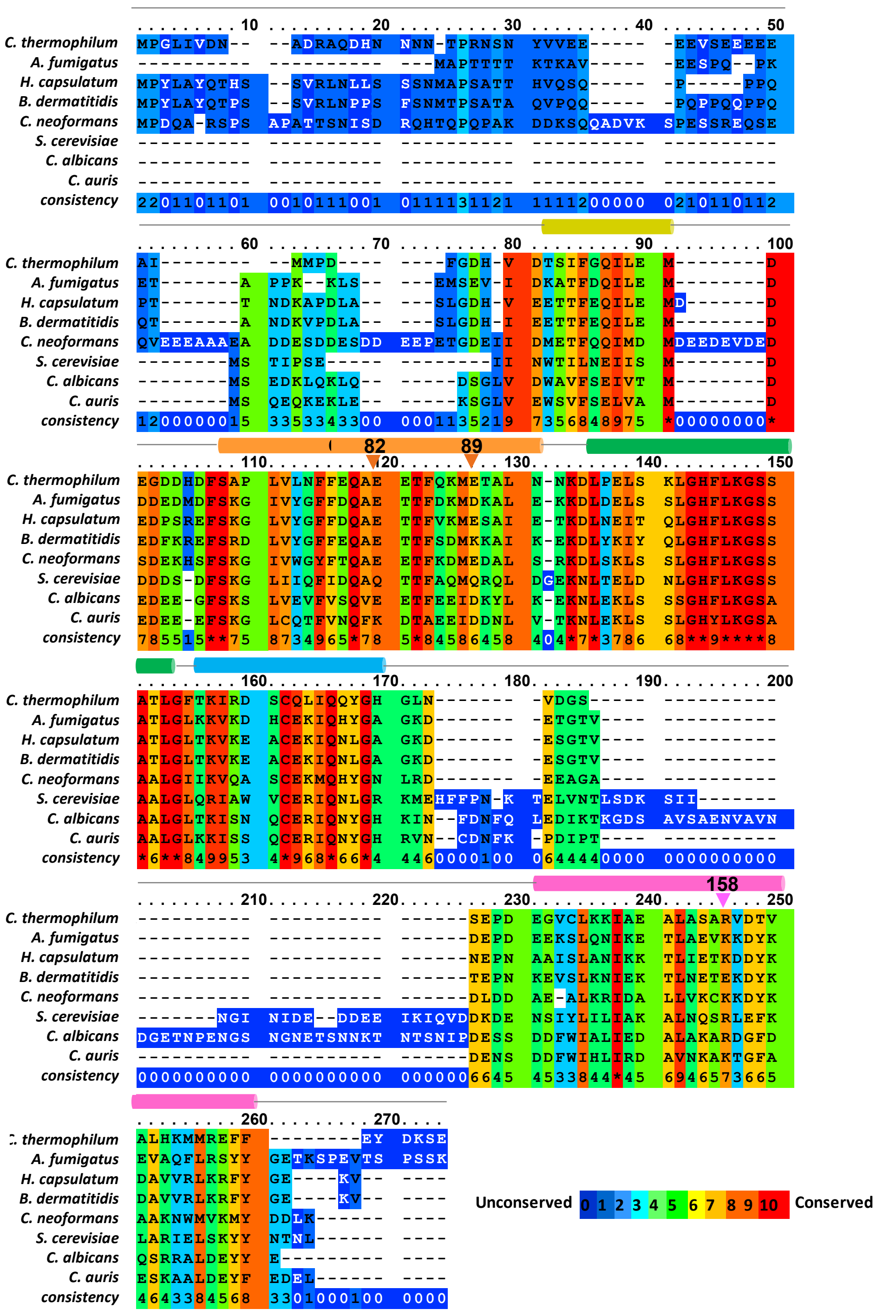

Supplementary Fig. 4. Structural comparison of REC-1:HPt complexes and sequence comparison of HPt in fungal species. a) Structural superposition of the complex REC_{hHK6}:Ct_HPt and REC_{Sc_Sln1}:Sc_Ypd1 in the presence of phosphomimetic BeF_3^- demonstrates similar spatial organization and location of catalytic residue and phosphomimetic. Changes in the conformation of loop β4α4 are observed b) Sequence alignment conservation of HPt/Ypd1 using the Praline server from *C. thermophilum, A. fumigatus, H. capsulatum, B. dermatitis, C. neoformans, S. cerevisiae, C. albicans* and *C. auris*. The α-helices composing, as the secondary structural elements, has been drawn over the sequence.

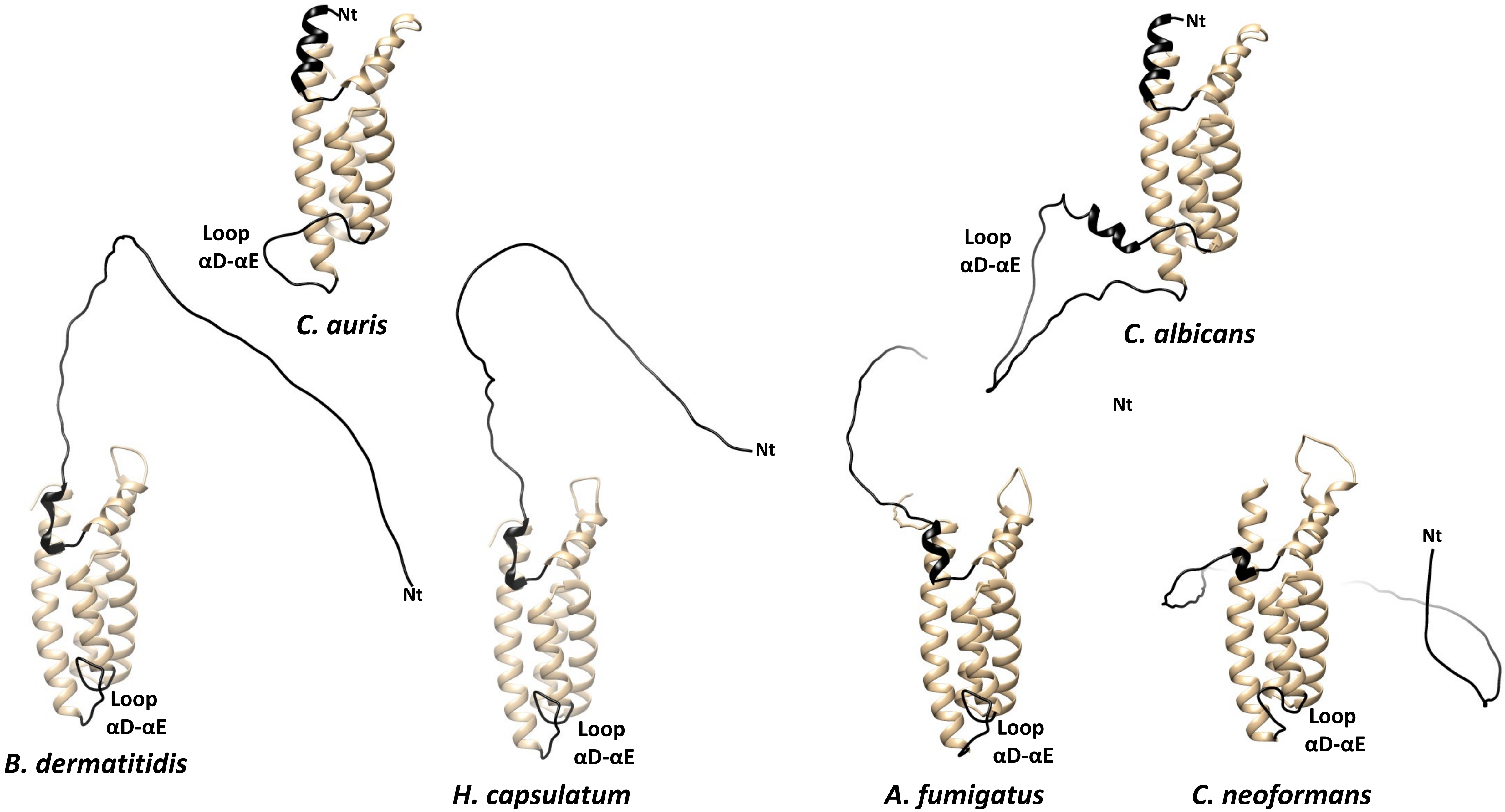

Supplementary Fig. 5. AlphaFold and RobeTTAFold model structures of HPt Ypd1 from human pathogenic fungi. AlphaFold model structures obtained for *A. fumigatus, H. capsulatum, B. dermatitidis, C. neoformans, S. cerevisiae, C. auris* and RobeTTAFold model structure for *C. albicans.* The N-terminal and loop αD-αE are colored in black.

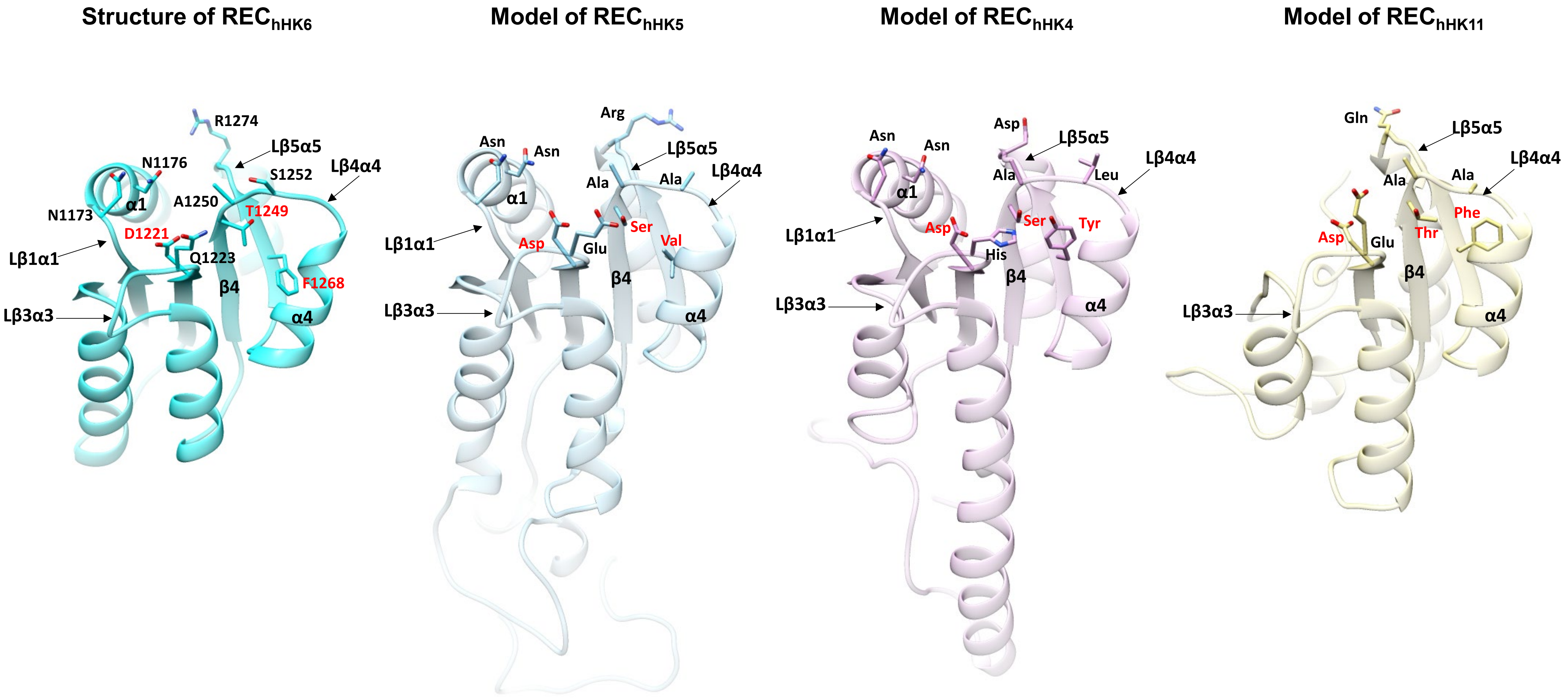

Supplementary Fig. 6. AlphaFold modelled structures of REC$_{hHK4}$, REC$_{hHK5}$ and REC$_{hHK11}$ compared to crystal structure of REC$_{hHK6}$. Some residues of REC$_{hHK6}$ involved in interaction with Ct_HPt are shown. Then, residues in the same position are located in the model structures of REC$_{hHK4}$, REC$_{hHK5}$ and REC$_{hHK11}$ (see Supplementary Table 2 as well). In REC$_{hHK6}$, the catalytic Asp (D1221), the conserved Thr in β4 (T1249) and Phe (F1268) in β5 are shown and labelled in red. Also, residues in the same position are located in the modelled structures and labelled in red.

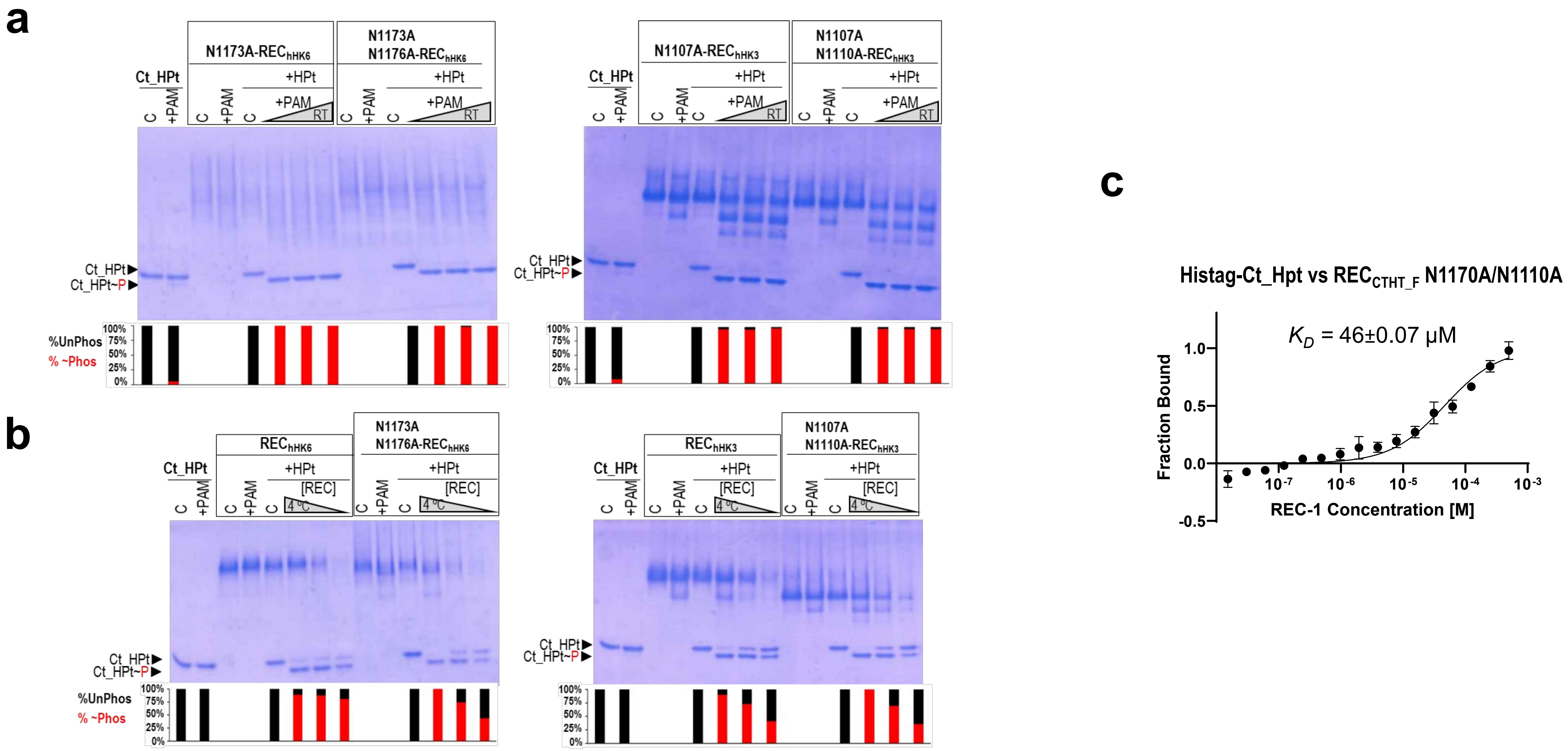

Supplementary Fig. 7. Phosphotransfer and interaction of REC$_{hHK6}$ and REC$_{hHK3}$ mutants with Ct_HPt. a) Native-gel phosphotransfer experiments phosphorylating WT and mutants of the conserved Asn residues located in α1 and loop β1-α1 (N1173A-REC$_{hHK6,}$ N1173A/N1176A-REC$_{hHK6,}$ and N1107A-REC$_{hHK3,}$ N1107A/N1110A-REC$_{hHK3)}$ with PAM for 30 min at 37 °C, then, allowed to cool down for 3 min to perform phosphotransfer at room temperature (RT) mixing with Ct_HPt for 0.5, 1 and 5 min. b) Native-gel phosphotransfer as in a) but mixing different ratios Ct_HPt:REC-1 domain (1:1, 1:0.5, 1:0.25) at 4 °C during 0.5 min. c) MST experiment using fluorescent labelled Histag-Ct_HPt as the probe and N1107A/N1110A-REC$_{hHK3}$ as the ligand. The lines containing “C” refers to the proteins without phosphodonor. Ratio 1:1 corresponds to ~20 µM:16 µM.

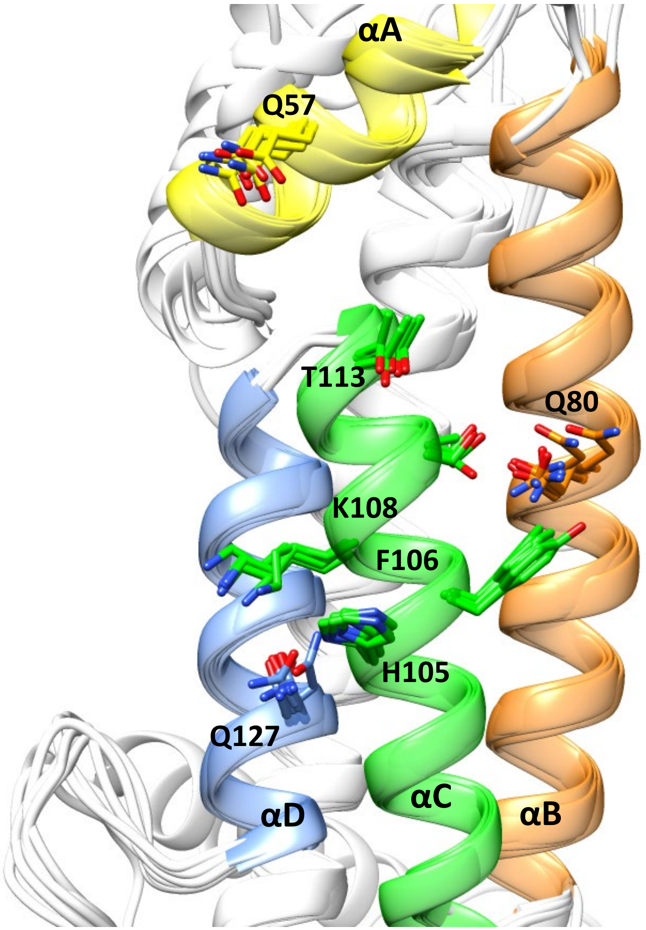

Supplementary Fig. 8. Conservation of the residues of HPt involved in interactions with REC-1 domain in fungi. Residues of Ct_HPt interacting with REC_{hHK6} are shown for *C. thermophilum* as well as for Ypd1 coming from *A. fumigatus, H. capsulatum, B. dermatitidis, C. neoformans, N. crassa, S. cerevisiae, C. albicans* and *C. auris.* The conservation of the residues in each protein is also indicated in Supplementary Table 3.

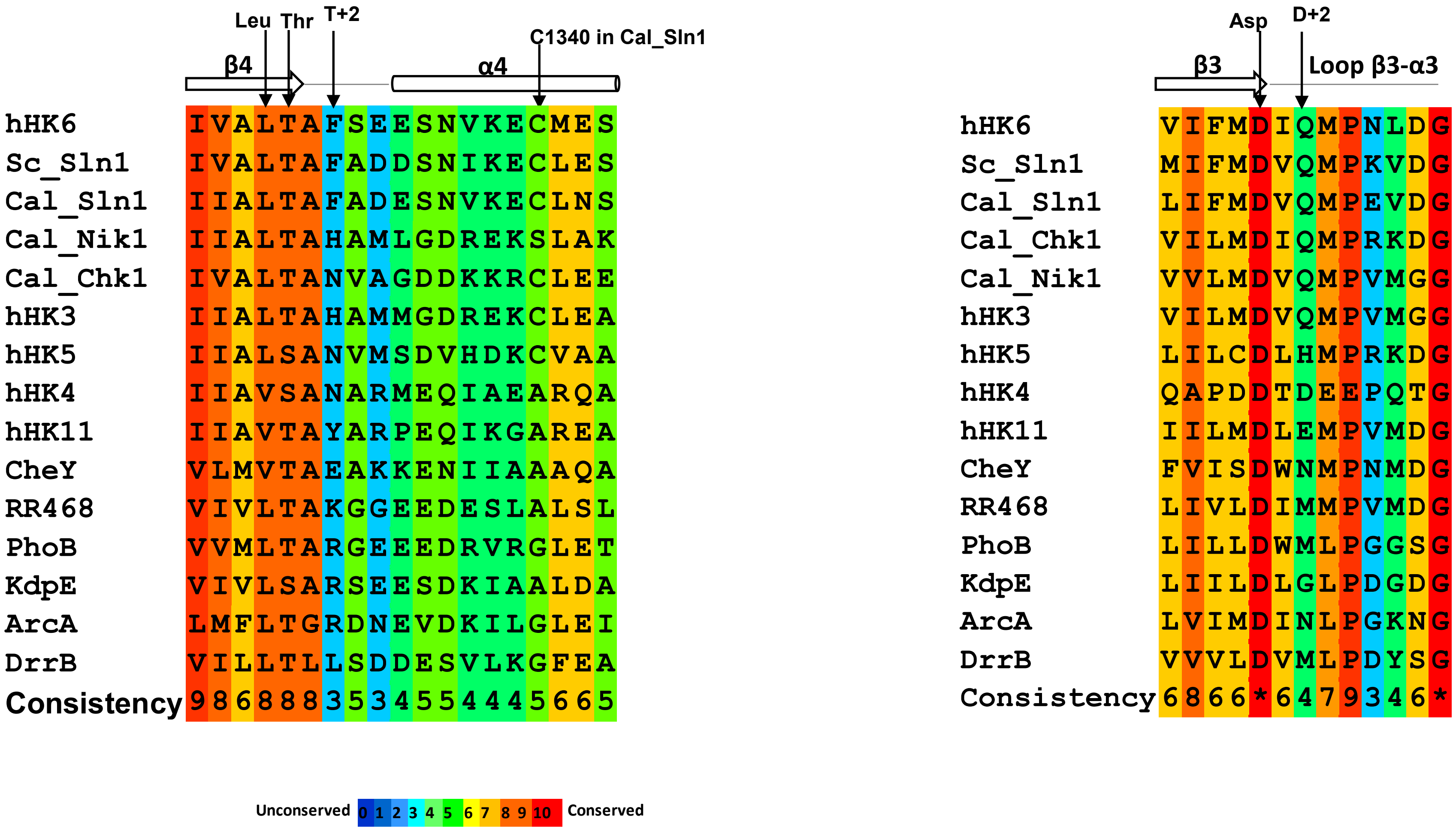

Supplementary Fig. 9. Sequence alignment from β4 to the end of α4 as well as from β3 to the end of loop β3-α3 across REC-1 domains of hHKs and bacterial REC domains. Sequence alignment and conservation from β4 to α4 of residues involved in the Leu-Thr switch for hHK Sln1 of *S. cerevisiae*, hHKs used in this study of *C. thermophilum*, hHKs of *C. albicans* (Sln1, Nik1 and Chk1) and bacterial RRs CheY, PhoB, KdpE, ArcA, DrrB and RR468. Sequence alignment for β3 and loop β3-α3 is also shown indicating the phosphorylatable Asp and other residue at D+2 position. The alignment and conservation has been obtained using the Praline server.

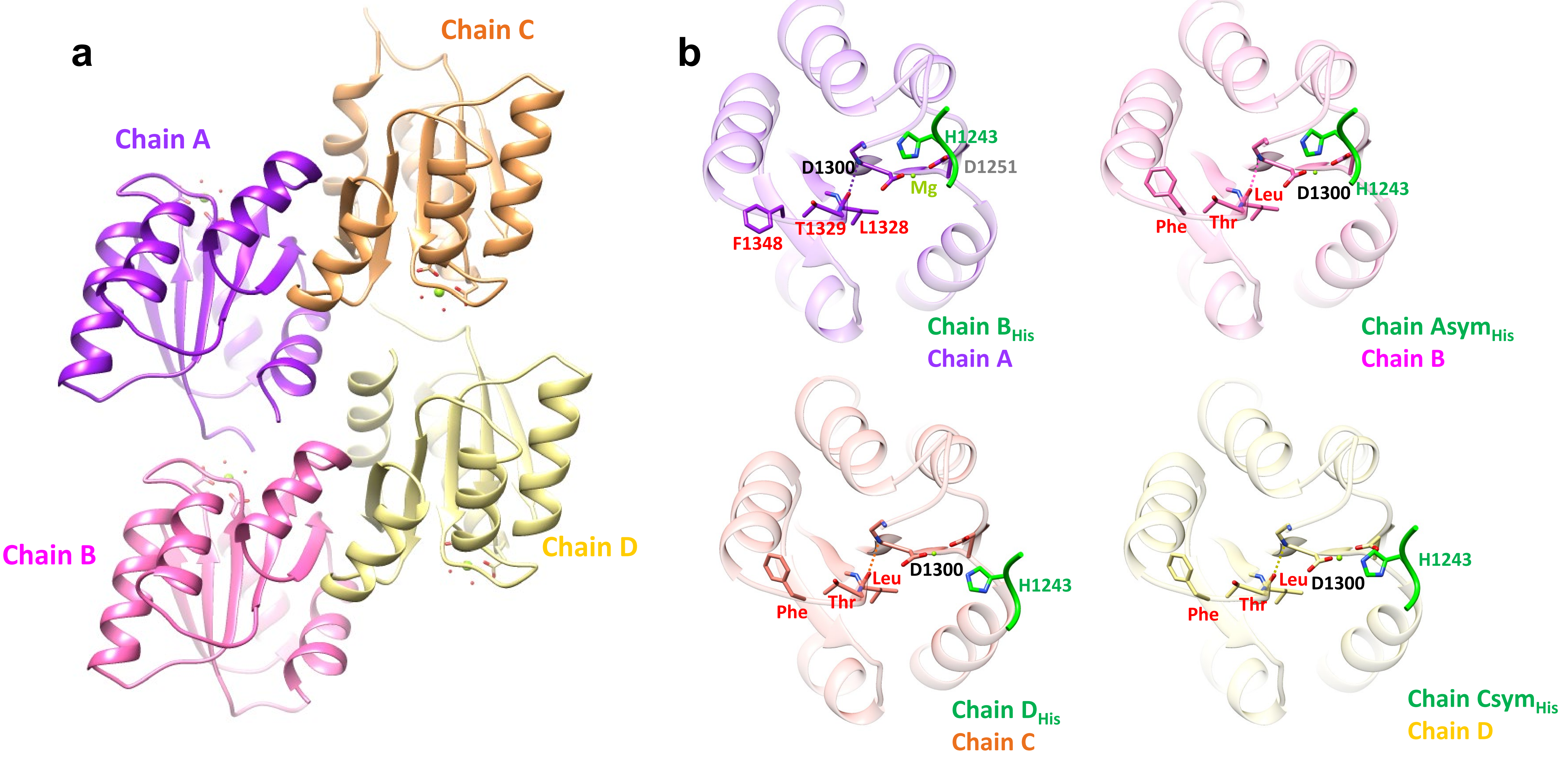

Supplementary Fig. 10. Structural features of REC$_{Cal_Sln1}$. a) Asymmetric unit of REC$_{Cal_Sln1}$ containing four molècules. The PISA server indicates the presence of two dimers A-C and B-D (buried area of ~1800 Å^2 and ΔG^{diss} of ~6 kcal/mol) b) Zoom of the active center for each molecule of REC$_{Cal_Sln1}$ highlighting the uncoupled residues of the Leu-Thr switch (L1328, T1329 and F1348, labelled in red), the catalytic Asp (D1300, labelled in black) bound to Mg^{2+} ion together with Asp1251 and the N-terminal H1243 (labelled in green) coming from another molecule.

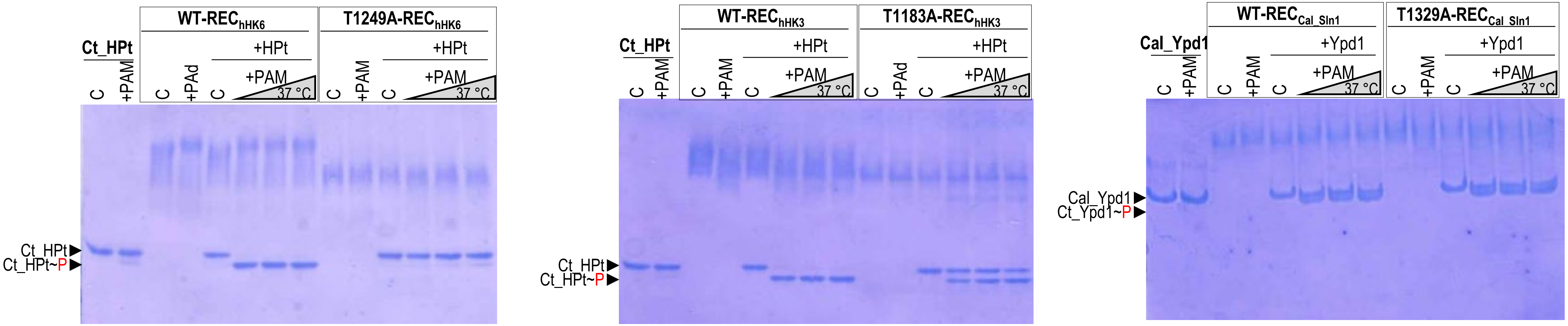

Supplementary Fig. 11. Effect of mutation at the conserved Thr in β4 in phosphotransfer. Phosphotransfer experiments from REC_{hHK6} WT and mutant T1249A to Ct_HPt, from REC_{hHK3} WT and mutant T1183A to Ct_HPt and REC_{Cal_Sln1} WT and mutant T329A to Cal_Ypd1 were performed. REC-1 domains were phosphorylated with PAM during 30 min at 37 °C and phosphotransfer to Ct_HPt or Ypd1 was performed during 0.5, 1 and 5 min at 37 °C. The lines containing “C” refers to the proteins without phosphodonor.

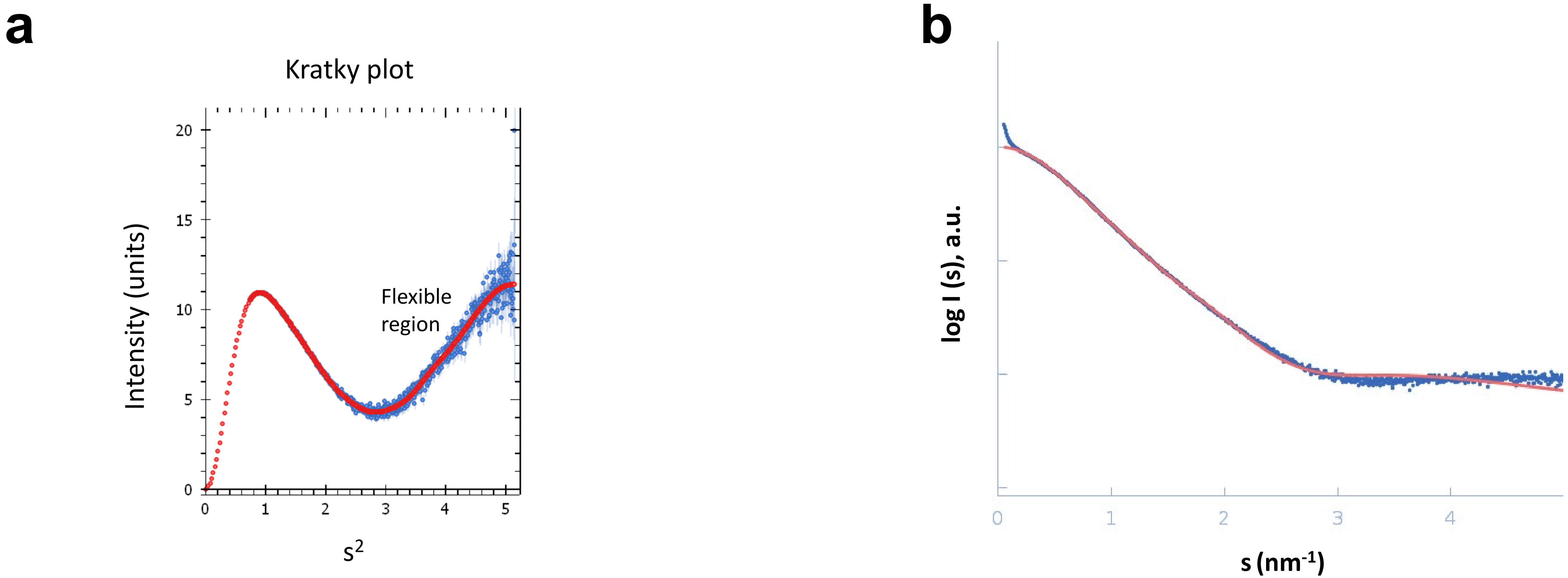

Supplementary Fig. 12. SEC-SAXS analysis obtained for the calculated Cal_Ypd1 envelope structure. a) Representation of the Kratky plot indicates the degree of flexibility in the structure. The peak indicates the presence of a globular region connected to a flexible region. b) Fitting of the model structure of Cal_Ypd1 obtained after SASREF on the SAXS experimental data. Curve extracted directly from the deposited data (code SASDSU5) in the SASBDB database.

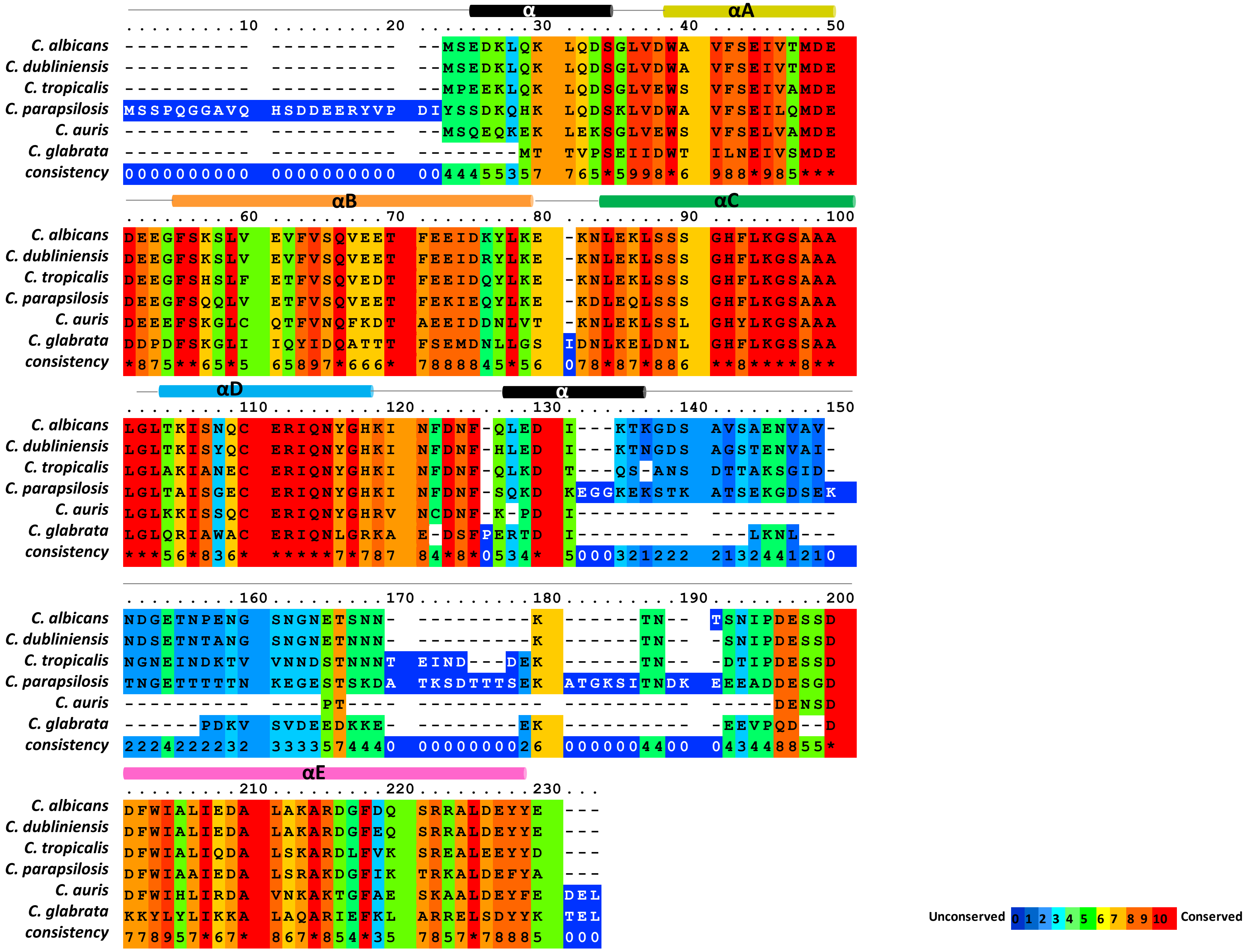

Supplementary Fig. 13. Sequence alignment of Ypd1 across the *Candida* genus using the Praline server.

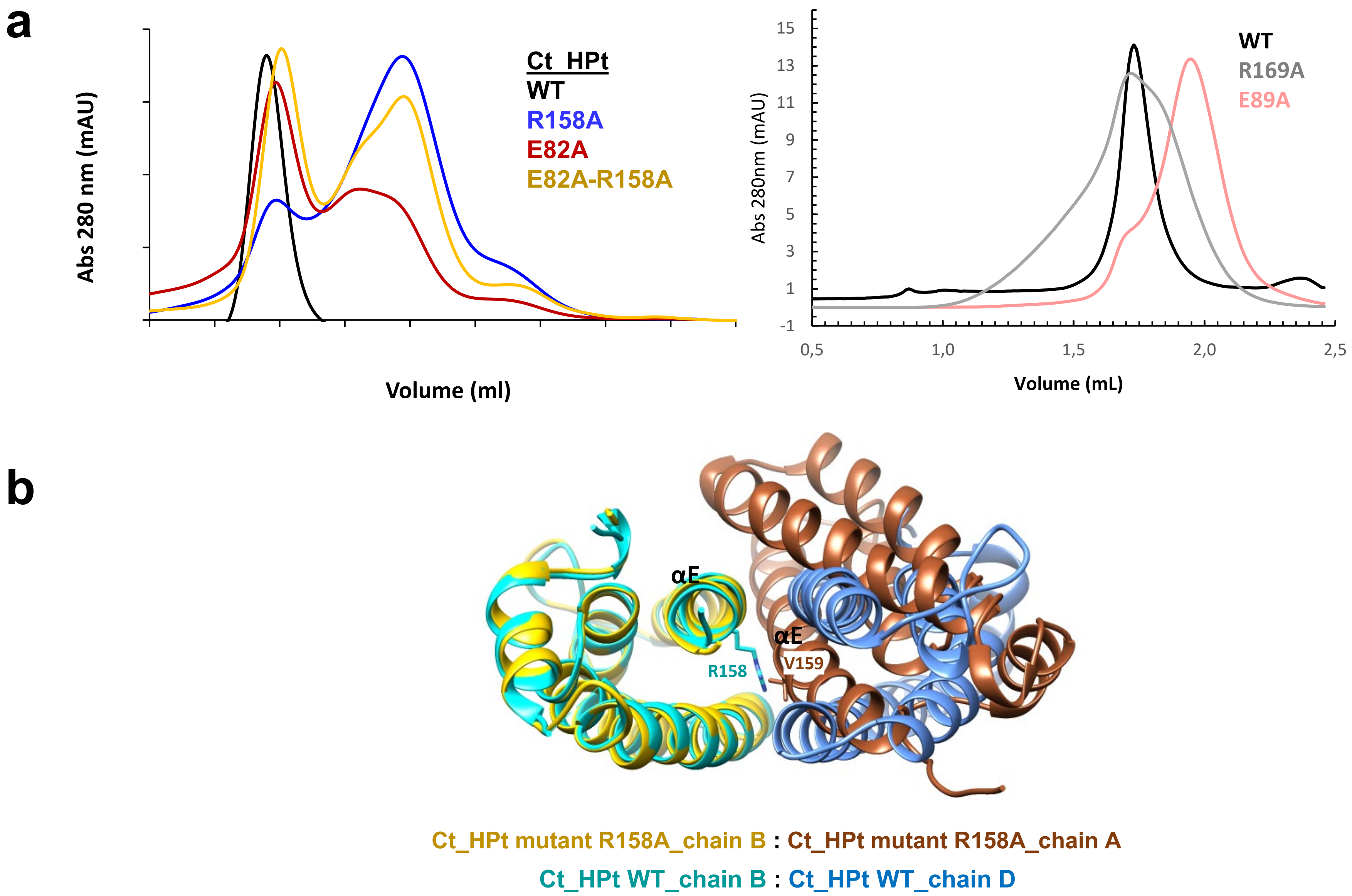

Supplementary Fig. 14. Size Exclusion Chromatography (SEC) of Ct_HPt mutants compared to WT. a) SEC analysis was performed using a ProSEC 11/30 6-600 HR (Generon) for the WT and mutants E82A, R158A and E82A-R158A. Meanwhile, SEC analysis was also performed for E89A and R169A with respect to WT using a Superdex® 200 Increase 3.2/300. The assays were performed in Buffer C. b) Superposition of crystal structure of Ct_HPt WT and mutant R158A. Two molecules of R158A mutant (in yellow and brown) found in the asymmetric unit of the crystal are drawn and the side chain of V159 in chain A is shown. Two molecules of superposed Ct_HPt WT (in blue hue) packed forming salt bridges are drawn and the side chain of R158A from chain B is shown.

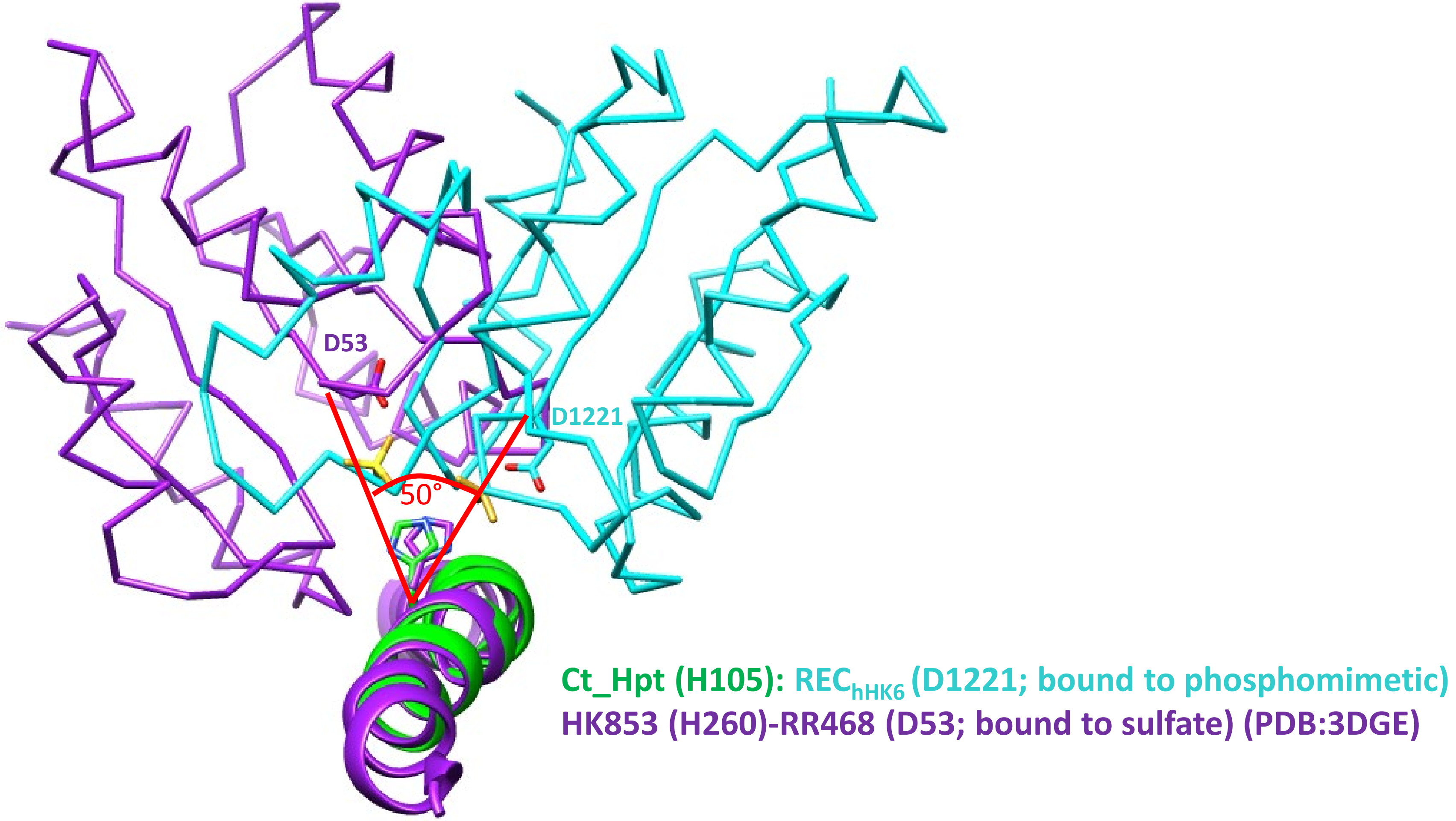

Supplementary Fig. 15. Structural comparison of complex REC_{hHK6}-BeF:Ct_HPt with complex HK853-RR468 (PDB:3DGE). The αC in Ct_HPt shows the phosphorylatable H105 (colored in green) and is superposed to α1 of HK853 containing the phosphorylatable H260 (colored in purple). Comparison between REC_{hHK6} and RR468 indicates that are docked differently to the α-helix containing the phosphorylatable His. In this way, the phosphorylatable D1221 bound to phosphomimetic in REC_{hHK6} (light blue) is 50° rotated (main chain distances) versus the phosphorylatable D53 in RR468 (in purple) bound to sulfate ion.

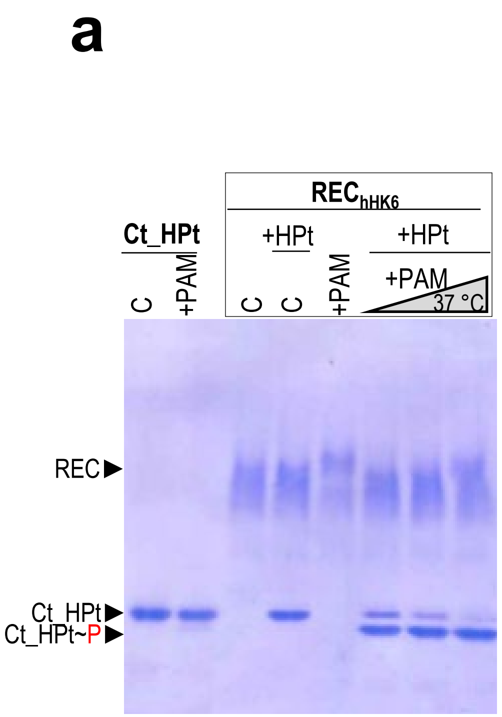

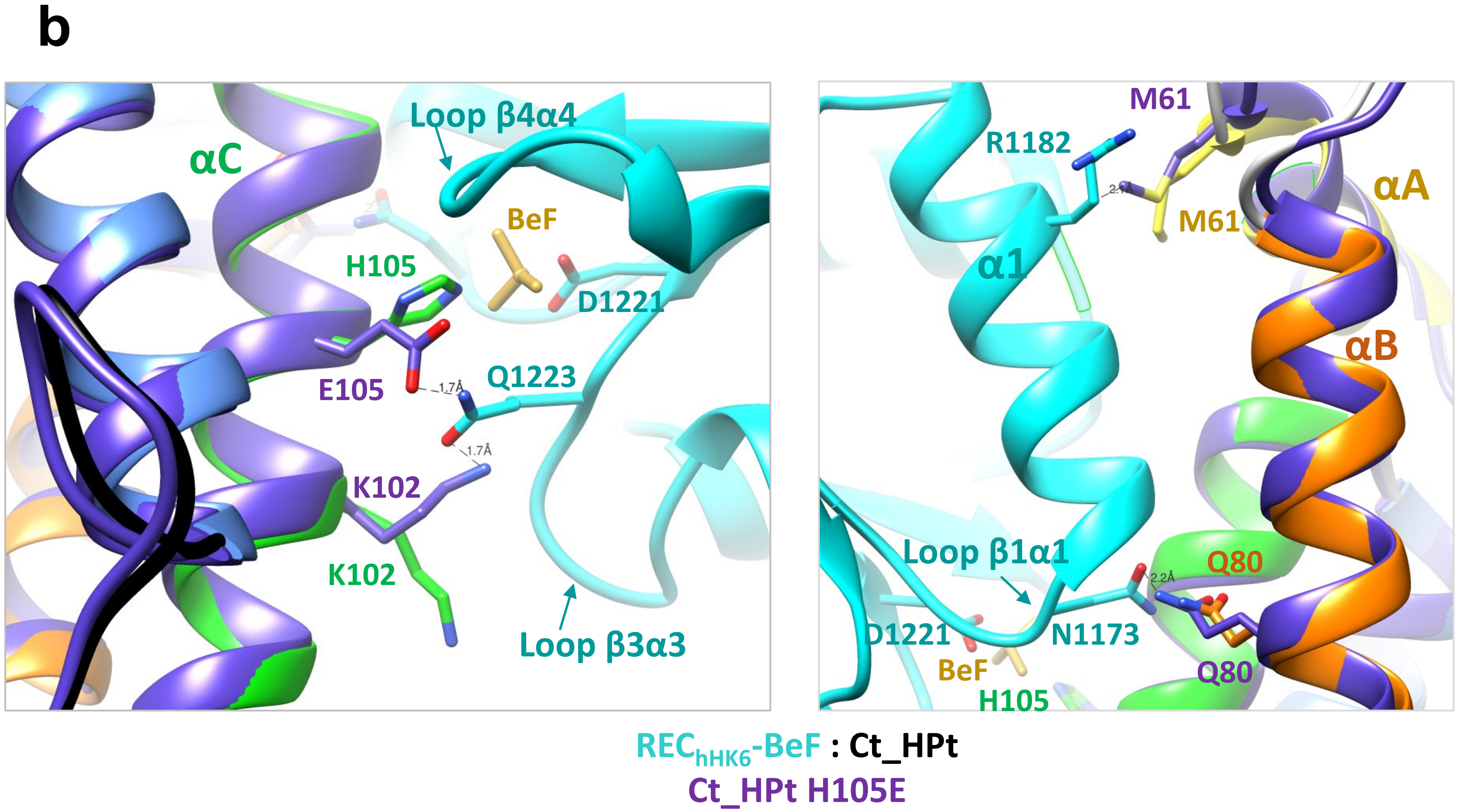

Supplementary Fig. 16. Role of Ct_HPt in reversibility. a) Native gel showing phosphotransfer upon incubation of Ct_HPt with REC$_{hHK6}$ from time 0 in the presence of PAM and further incubation at 0.5, 1 and 5 min. b) Structure of phosphomimetic mutant Ct_HPt H105E superposed with the Ct_HPt WT in the complex structure REC$_{hHK6}$:Ct_HPt. Left panel shows clashes between the superposed Ct_HPt H105E mutant with residue Q1223 in loop β3-α3. Right panel shows clashes between Ct_HPt H105E mutant and N1173 and R1182. The lines containing "C" refers to the proteins without phosphodonor.

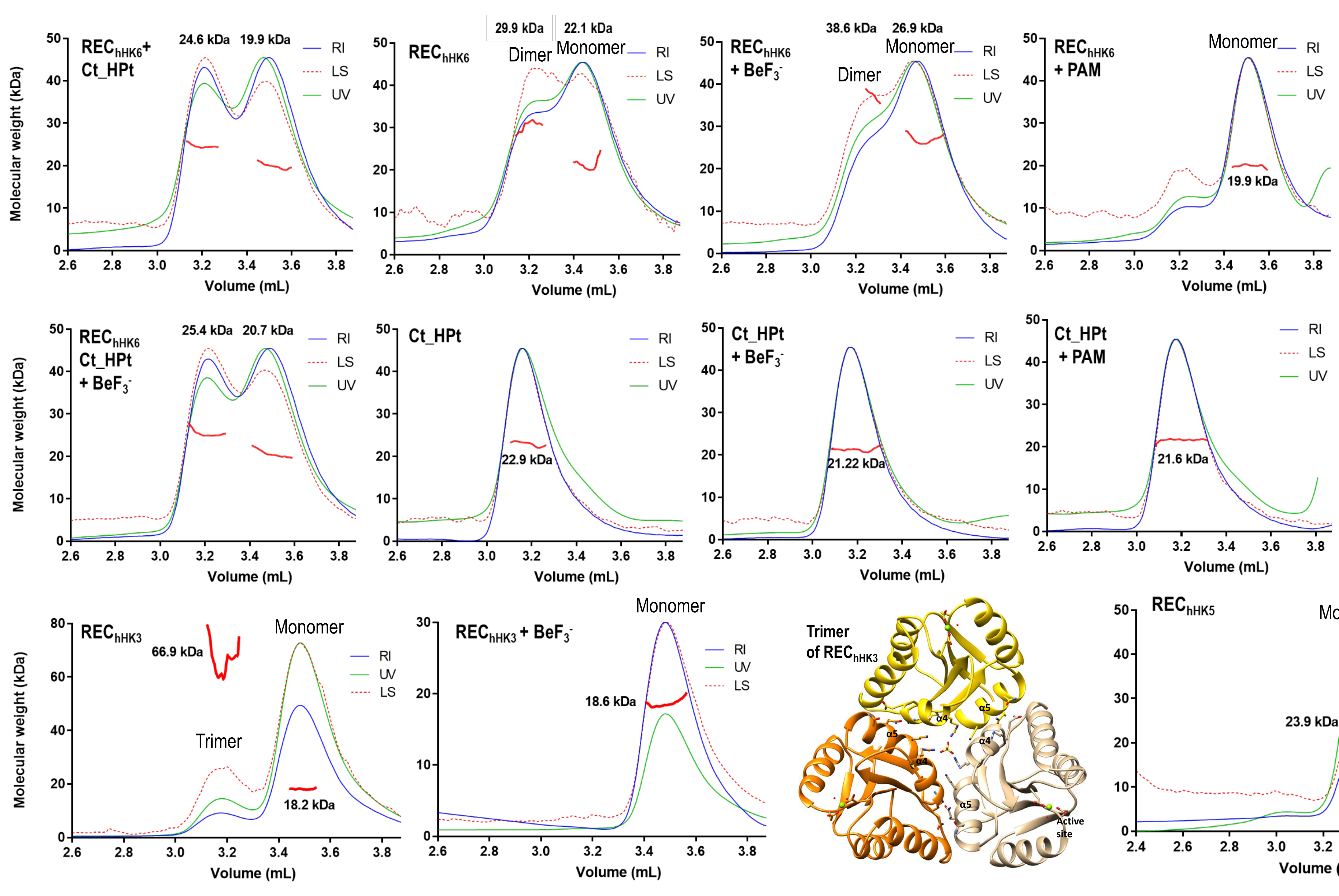

Supplementary Fig. 17. SEC-MALS analysis. Upper panel shows REC$_{hHK6}$ mixed with Ct_HPt, REC$_{hHK6}$ alone and with phosphomimetic or phosphodonor PAM. Middle panel shows REC$_{hHK6}$ mixed with Ct_HPt and phosphomimetic as well as Ct_HPt alone or mixed with phosphomimetic and phosphodonor. Bottom panel shows REC$_{hHK3}$ alone and mixed with phosphomimetic, REC$_{hHK5}$ and the crystallographic trimer of REC$_{hHK3}$ detected by the PISA server. Peaks for trimer, dimer or monomer in REC-1 domains have been labelled.
